# Photopatternable PEDOT:PSS Hydrogels for High‐Resolution Photolithography

**DOI:** 10.1002/advs.202414834

**Published:** 2025-03-24

**Authors:** Wen Wang, Jingcheng Liu, Hai Li, Yi Zhao, Rongtai Wan, Qiaobo Wang, Jingkun Xu, Baoyang Lu

**Affiliations:** ^1^ Jiangxi Provincial Key Laboratory of Flexible Electronics Flexible Electronics Innovation Institute Jiangxi Science & Technology Normal University Nanchang 330013 P. R. China; ^2^ School of Chemical and Material Engineering Jiangnan University Wuxi 214122 P. R. China; ^3^ Robotics Institute and State Key Laboratory of Mechanical System and Vibration School of Mechanical Engineering Shanghai Jiao Tong University Shanghai 200240 P. R. China; ^4^ School of Water Resources & Environmental Engineering East China University of Technology Nanchang 330013 P. R. China

**Keywords:** bi‐continuous phase, conducting polymer hydrogels, high resolution, PEDOT:PSS, photolithography

## Abstract

Conducting polymer hydrogels have been extensively explored toward diverse applications like bioelectronics and soft robotics. However, the fabrication resolution of conducting polymer hydrogels by typical techniques, including ink‐jet printing, 3D‐printing, etc., has been generally limited to >10 µm, significantly restricting rapid innovations and broad applications of conducting polymer hydrogels. To address this issue, a photosensitive biphasic conducting polymer hydrogel (PB‐CH) is rationally designed and synthesized, comprising poly(3,4‐ethylenedioxythiophene):poly(styrene sulfonate) (PEDOT:PSS) as the conductive phase and a light‐sensitive matrix as the mechanical phase. The formation of phase‐separated structures within PB‐CH preserves the integrity of the conductive channels during the photoinitiated cross‐linking. This minimizes the conductivity loss, a common limitation in similar materials. Remarkably, the resultant PB‐CH exhibits a combination of excellent electrical conductivity (≈30 S cm^−1^), robust mechanical performance (tensile strain up to 50%), and high photopatternability. A detailed investigation of the photolithography process identifies key technological parameters that enable high‐resolution patterning of 5 µm. By simultaneously maintaining processability, conductivity, and mechanical flexibility, this PB‐CH represents an ideal candidate for advanced flexible electronic applications, offering a new technique to fabricating high‐performance conducting polymer hydrogels.

## Graphical abstract

A photosensitive biphasic conducting polymer hydrogel (PB‐CH) is rationally designed with PEDOT:PSS as the conductive phase and a light‐sensitive matrix as the mechanical phase. Such a PB‐CH offers a unique combination of excellent conductivity (≈30 S cm⁻¹), robust mechanical performance (with stretchability up to 50%), and high photopatternability with feature sizes down to 5 µm, advancing precision in flexible electronics fabrication.

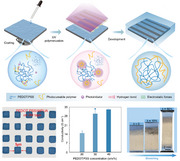

## 1 Introduction

Conducting polymer hydrogels, which are polymer networks with tissue‐mimicking mechanical properties and excellent electrical properties, have emerged as a versatile class of materials in biomedical devices, flexible electronic devices, soft robotics, and other fields.^[^ ^1^, ^2^, ^3^, ^4^, ^5^ ^]^ Among them, hydrogels based on poly(3,4‐ethylenedioxythiophene):poly(styrene sulfonate) (PEDOT:PSS) are particularly notable due to their unique combination of electrical conductivity and electrochemical stability in physiological environments.^[^ ^6^, ^7^, ^8^, ^9^, ^10^, ^11^ ^]^ To optimize the performance of PEDOT:PSS hydrogels, researchers have employed various strategies, including synthetic approaches,^[^ ^12^, ^13^ ^]^ additive engineering,^[^ ^14^, ^15^, ^16^ ^]^ and post‐treatment designs.^[^ ^6^, ^17^, ^18^, ^19^ ^]^ These methods have significantly enhanced the tunability of PEDOT:PSS hydrogels in terms of composition, structure, and functionality.^[^ ^20^, ^21^ ^]^ Such advancements have enabled these hydrogels to function effectively as bioelectronic components, especially in demanding applications such as neural recording, electrical stimulation, and real‐time physiological monitoring.^[^ ^17^, ^22^, ^23^, ^24^ ^]^ Despite significant progress has been achieved in materials synthesis and performance development, there still remains a critical need for reliable, high‐resolution patterning methods to fabricate PEDOT:PSS‐based hydrogels on flexible substrates. To meet the growing demands for mechanical flexibility, miniaturization, and precise device configurations in next‐generation bioelectronics, patterning at resolutions down to 10 µm or even sub‐micron levels is essential. The development of techniques that can achieve such fine resolutions is crucial for enhancing the adaptability and functionality of these hydrogels in applications that require conformal contact with soft tissues, thereby driving innovations in bioelectronics and soft robotics.

An ideal patterning method for PEDOT:PSS‐based hydrogels should meet three key criteria: i) it should be applicable to PEDOT:PSS to achieve both excellent conductivity and tissue‐like mechanical properties, ii) it should be fully compatible with direct patterning on flexible substrates without requiring additional transfers, and iii) it should be capable of producing high‐resolution patterns as well as complex structural patterns. However, current methods fail to meet all of the above requirements simultaneously. For instance, laser direct writing induces phase separation of PEDOT:PSS due to the high energy density involved. This results in a significant reduction in water content within the hydrogel, thereby increasing Young's modulus and diminishing tensile properties. Ultimately, this leads to reduced compatibility with biological tissues, limiting its practical application in biomedical devices.^[^ ^16^ ^]^ Furthermore, techniques like 3D‐printing^[^ ^7^, ^8^, ^10^, ^25^ ^]^ and screen printing^[^ ^24^, ^26^ ^]^ can pattern PEDOT:PSS‐based conducting polymer hydrogels under mild conditions, but achieving high resolution (e.g., patterns smaller than 10 µm) remains challenging. The challenges arise from the complexity of ink formulation,^[^ ^7^ ^]^ viscosity optimization,^[^ ^8^ ^]^ and subsequent post‐processing steps^[^ ^10^, ^27^ ^]^ required to transform the printed ink into a functional hydrogel. For instance, Feng et al. employed a two‐step cross‐linking approach involving extensive solvent washing, drying at 130 °C, and subsequent swelling to achieve a functional PEDOT:PSS hydrogel.^[^ ^10^ ^]^

In contrast, photolithography offers the ability to produce high‐resolution patterns in a parallel and scalable manner.^[^ ^28^, ^29^, ^30^ ^]^ However, current photolithography approaches that directly mix PEDOT:PSS with photosensitive polymers often overlook the issue of phase separation, which is crucial for maintaining high conductivity. Although the photo‐crosslinking process can enhance the mechanical properties of the material, it typically results in low electrical conductivity (<1 S cm^−1^).^[^ ^31^, ^32^ ^]^ For example, David et al. reported that blending PEDOT:PSS with photosensitive polymer poly(ethylene glycol) diacrylate (PEGDA) significantly reduced its conductivity to 0.1 S cm^−1^.^[^ ^31^ ^]^ This reduction was attributed to the insulating nature of the matrix, which disrupted the conductive pathways. Similarly, Luciano et al. demonstrated that the addition of PEGDA‐based photosensitive monomers reduced the uniformity of the conductive PEDOT:PSS network, resulting in a poor electrical performance of 0.05 S cm^−1^.^[^ ^32^ ^]^ Thus, while photolithography excels in precise pattern formation, maintaining the desired electrical and mechanical properties of PEDOT:PSS hydrogels simultaneously remains a significant challenge.

Herein, we have rationally designed and synthesized a photosensitive biphasic conducting polymer hydrogel (PB‐CH), comprising PEDOT:PSS as the conductive phase and a light‐sensitive matrix as the mechanical phase, optimized for direct photolithography. Unlike previous works that focus on blending materials without addressing the trade‐offs between conductivity and mechanical properties, our approach specifically targets the preservation of conductive pathways during the photopolymerization process. This is achieved through the phase‐separated structure of PB‐CH, which minimizes disruption to the PEDOT:PSS conductive network while improving mechanical flexibility. Consequently, the PB‐CH simultaneously achieves excellent conductivity (≈30 S cm^−1^), exceptional electrochemical stability, and a tensile strain of 50% while also being highly compatible with high‐resolution photolithography, achieving feature sizes as small as 5 µm. This unique combination of conductivity, mechanical property, and processability positions PB‐CH as an ideal candidate for advanced flexible electronics.

## 2 Results and Discussion

### 2.1 The design of Photosensitive Biphasic Conducting Polymer Hydrogel (PB‐CH)

**Figure 1:**
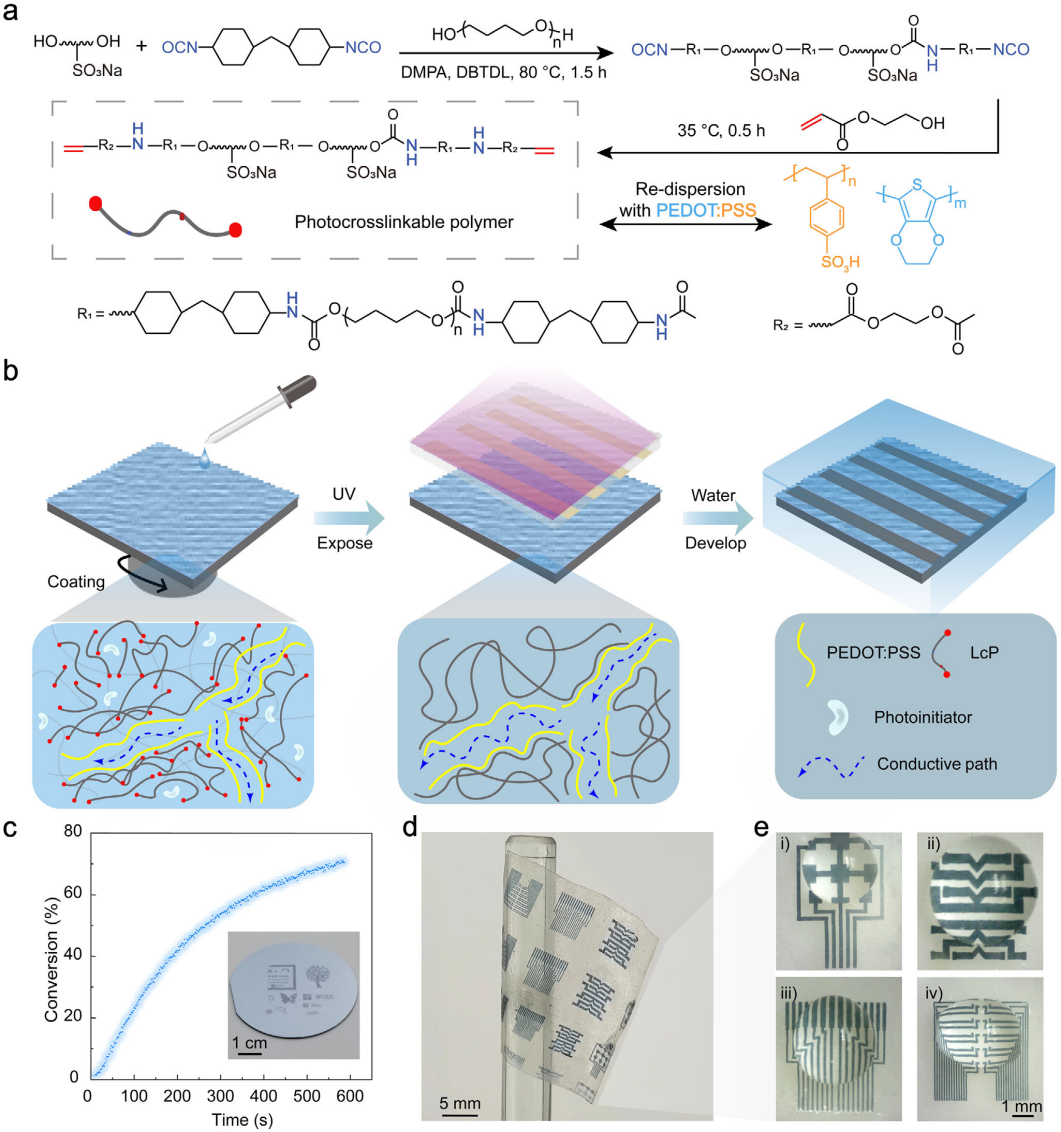
Schematic illustration of direct photolithography processing of conducting polymer hydrogel. a) Synthesis of PB‐CH. b) Schematic illustration of the direct photolithography process for PB‐CH involves selective exposure via UV irradiation and a photomask, followed by development in DI water. c) UV‐induced photopolymerization of PB‐CH monitored by real‐time FTIR analysis, which shows the double bond conversion as a function of irradiation time. The inset is a photograph of patterned PB‐CH on a 2‐inch wafer. d, e) Patterned PB‐CH electrode arrays with different shapes and sizes on a flexible substrate.

The photosensitive biphasic conducting polymer hydrogel (PB‐CH) is engineered to integrate electrical conductivity and mechanical flexibility for advanced applications. This design employs a photosensitive approach based on the photochemistry of a light‐curing polymer (LcP), where a photoinitiator releases free radicals under UV irradiation (365 nm), initiating the polymerization of long‐chain LcP (**Figure** **1**a). PEDOT:PSS is selected as the conductive phase due to its stable electrical properties, while a hydrophilic polyurethane‐based LcP serves as the mechanical phase, enhancing the hydrogel's mechanical resilience. The phase separation of hydrophilic polyurethane within the mixed solvent, due to its differential solubility in ethanol and water, enables effective structural formation, as demonstrated in previous work.^[^ ^27^ ^]^ Upon UV exposure, photochemical reactions are localized in the irradiated regions, forming a robust 3D network through hydrogen bonding and electrostatic interactions with PEDOT:PSS. This network solidifies into a hydrogel structure during development. The biphasic structure design minimizes disruption to the conductive channels within PEDOT:PSS, thus preserving its electrical properties, a crucial factor for bioelectronic applications. The fabrication process is carried out in three key steps: i) coating a photolithographable hydrogel precursor solution (consisting of PEDOT:PSS, LcP, and photoinitiators) onto a flexible substrate and drying, ii) UV irradiation of selected areas by a predesigned photomask, and iii) development using water as a developer to remove the unexposed area pattern (Figure 1b; Figure S5, Supporting Information). Notably, this strategy ensures that the conductive network within the PB‐CH structure remains largely intact, avoiding the loss of conductivity typically observed in conventional cross‐linking methods.

The LcP, designed as a long‐chain polymer with reactive double bonds, exhibits negative photoresist properties in combination with PEDOT:PSS, enabling selective patterning. To further elucidate the UV polymerization of LcP in the PB‐CH system, Fourier transform infrared (FT‐IR) analysis is performed concurrently, with the double‐bond conversion determined by tracking the evolution of the C═C peak (Figure S3, Supporting Information).^[^ ^33^, ^34^ ^]^ The double bond conversion of LcP increased rapidly initially, plateauing as the irradiation time progressed, with the final conversion rate reaching 71.3% (Figure 1c). Building upon the efficient mechanism of double‐bond polymerization, we employ a photomask for precise patterning. Through the design of the photomask, various structures of PB‐CH electrode arrays can be fabricated (Figure 1d,e). This entire process obviates the necessity for conventional photoresists, organic solvents, or their chemical constituents, which might compromise the properties of PEDOT:PSS.

### 2.2 Characterization and Interaction Analysis of PB‐CH

**Figure 2:**
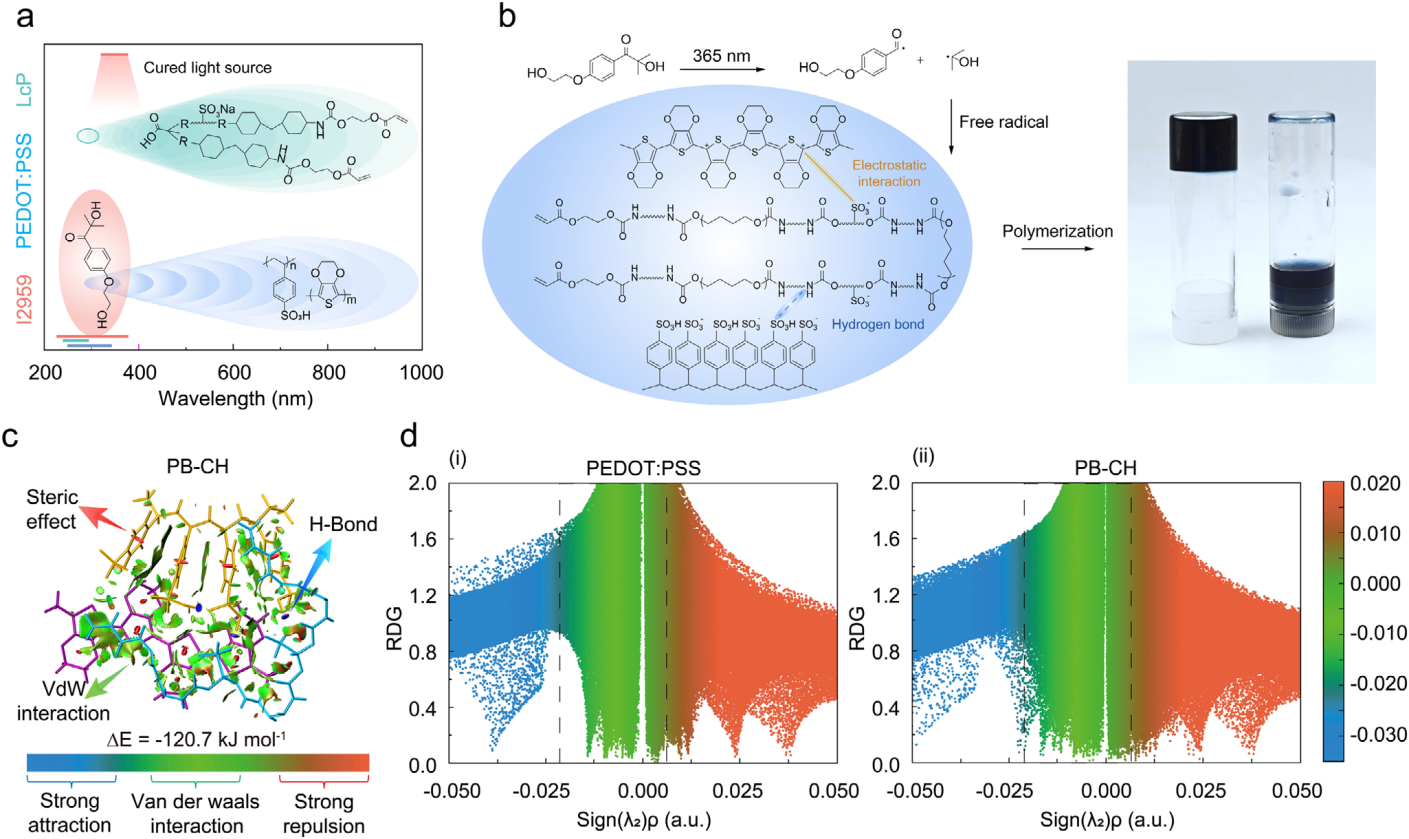
Direct photolithographic curing mechanism of PB‐CH. a) The maximum absorption peak of the UV spectrum of LcP (violet), PEDOT:PSS (blue), and Irgacure 2959 (pink). LcP shows strong absorption at ≈294 nm, differing from the excitation light source used. b) Polymerization mechanism in PB‐CH. The electrostatic interactions and hydrogen bonds are formed between the chemical components (PEDOT, PSS and LcP). c) Gradient isosurfaces for the bond of PB‐CH of reduced density gradient (RDG). The color bar indicates that blue, green, and red correspond to robust interactions, such as hydrogen bonding and electrostatic interaction, van der Waals interaction, and significant repulsion, such as *π–π* stacking and cation‐π interaction, respectively. d) Scatter plots of the RDG versus the electron density multiplied by sign of the second Hessian eigenvalue for i) PEDOT:PSS and ii) PB‐CH.

PEDOT:PSS commonly exists as an aqueous suspension containing oligomeric PEDOT in the cationic oxidation state, electrostatically bound to PSS.^[^ ^6^, ^35^, ^36^ ^]^ Nonetheless, the high acidity of PSS in PEDOT:PSS formulations may cause destabilization when combined with other components. To evaluate this, we first investigated the effects of incorporating commonly used monomers, such as acrylic acid (AA), acrylamide (AM), and 2‐hydroxyethyl methacrylate (HEA), without thermal initiators or cross‐linkers. Upon drying at 50 °C, the film maintained structural integrity in an aqueous environment without breaking down into microgels, as shown in Figure S6 (Supporting Information). Figure 2 illustrates the chemicals used to formulate this UV‐curable PB‐CH precursor solution, along with the bonding mechanism between PEDOT:PSS and LcP. The designed and synthesized LcP exhibits strong UV absorption near 294 nm (Figure S7, Supporting Information), with minimal overlap with the absorption peak of the commonly used commercial water‐soluble photoinitiator Irgacure 2959 (I2959) (**Figure** **2**a).^[^ ^37^ ^]^ In the PB‐CH precursor, I2959 initiates polymerization via a two‐step photoinitiation process (Figure 2b): multiphoton absorption followed by the generation of free radicals (hydroxyethyl ether benzoyl peroxide radical and α‐hydroxyisopropyl free radical).

To elucidate the interactions between PEDOT, PSS, and LcP, we employed density functional theory (DFT) calculations to model the interactions and spatial distributions between the components (Figure 2c). 3D visualization of the isosurfaces, using colored scale bars (blue–green–red), distinguishes different weak interactions. Additionally, a reduced density gradient (RDG) scatter plot (Figure 2d) enables qualitative analysis of the interaction differences between PEDOT:PSS and PB‐CH. The peak electron density in the blue region (symbol (λ2)ρ from −0.05 to −0.02) increases significantly, suggesting that the introduction of LcP enhances the internal interactions within PEDOT:PSS (Figure S8, Supporting Information). Subsequently, the calculation of the interaction energy of PB‐CH yields a value of −120.7 kJ mol^−1^. Although this value is notably lower than that of PEDOT:PSS /PAM (polyacrylamide),^[^ ^38^ ^]^ PB‐CH does not undergo polymerization during the soft‐bake process, enabling subsequent photopolymerization for selective cross‐linking. It is important to note that the bonding between PEDOT:PSS and LcP primarily occurs through electrostatic attractions between the π‐conjugated PEDOT chains and the negatively charged PSS chains and LcP chains, as well as hydrogen bonding between the sulfonic acid groups of the PSS chains and the amine groups of LcP. These findings support the formation of phase‐separated structures within PB‐CH, a feature that underscores its potential for high‐precision hydrogel processing via photolithography. This biphasic structure highlights the advantages of PB‐CH in achieving superior performance in hydrogel patterning applications.

### 2.3 Structural and Adhesion Analysis of PB‐CH

**Figure 3:**
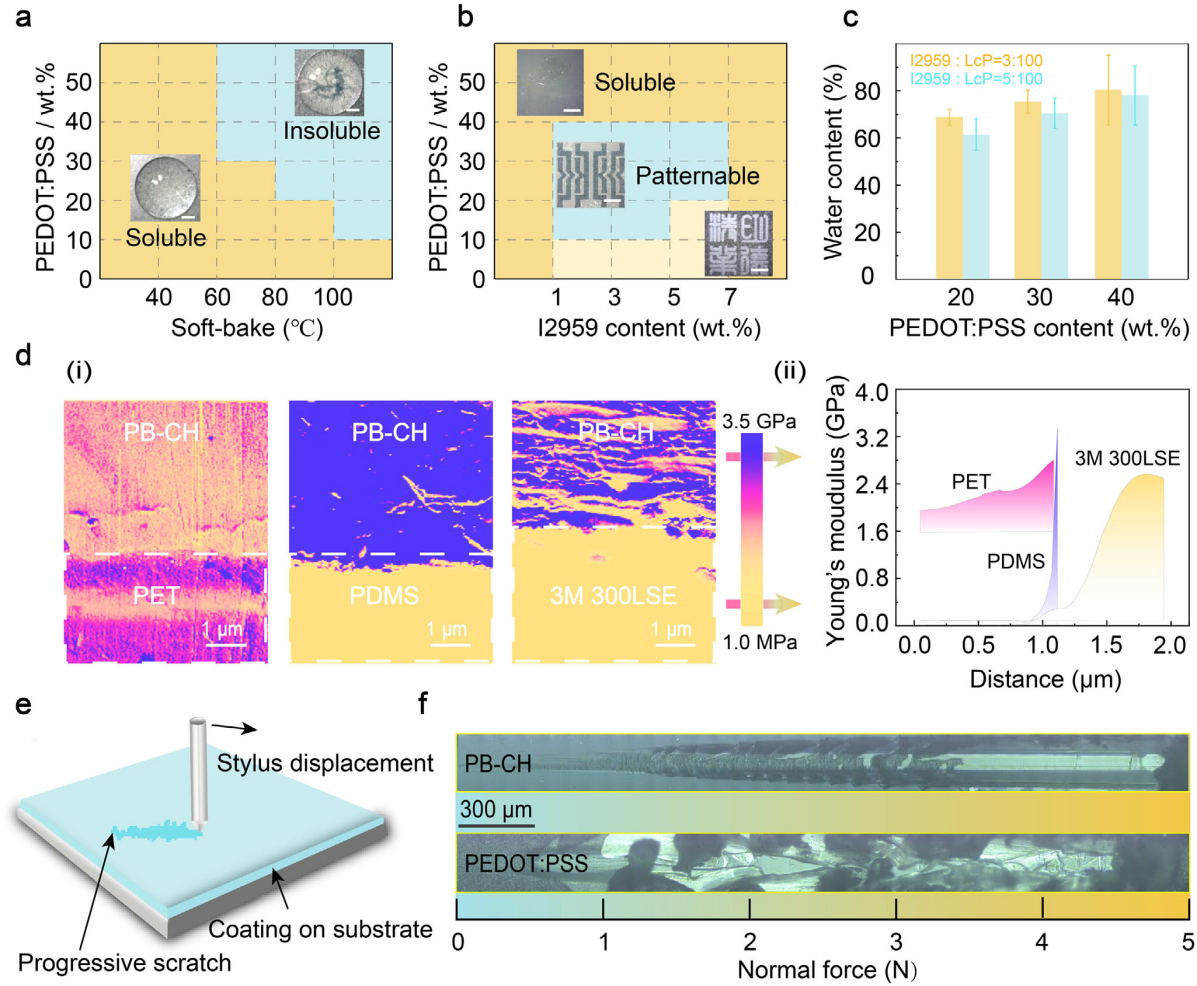
Direct photolithography printability of PB‐CH inks on flexible substrates. a) Phase diagram showing the precursor compositions that undergo phase separation during soft‐bake, with the weight ratio of PEDOT:PSS to PB‐CH ranging from 10 to 50 wt.%. Orange (background): the precursor did not phase separate during soft‐bake and remained soluble in DI water; Blue (background): the precursor phase separated during soft‐bake and became insoluble in DI water. b) Phase diagram illustrating the photopolymerizable regions of the precursor compositions undergoing photopolymerization with I2959 concentrations ranging from 1 to 5 wt.%. The precursor was soft‐baked at 50 °C and then selectively exposed through a photomask before being immersed in DI water. Orange (background): the exposed and unexposed areas of the precursor both dissolve in DI water; Blue (background): the exposed regions of the precursor can replicate the patterns of the photomask, while the unexposed regions dissolve in DI water. c) Dependence of water content of sample with different content of I2959. d) AFM characterization of the modulus gradient across the interfaces between PB‐CH and flexible substrates. i) modulus mapping, where the lower‐modulus region corresponds to the PB‐CH film (top) and the higher‐modulus region corresponds to the flexible substrate (bottom); ii) fitted interfacial profiles derived from the AFM images. e) The schematic diagram of the scratch test. f) The images of scratch test of PB‐CH coating and the PEDOT:PSS coating for comparative analysis. Scale bars, 1 mm (a,b).

In an aqueous environment, the structure of PEDOT:PSS is envisioned as comprising colloidal particles with a hydrophilic PSS shell surrounding a hydrophobic PEDOT core.^[^ ^39^, ^40^ ^]^ When dried at elevated temperatures, PEDOT:PSS undergoes phase separation, forming a uniformly distributed network structure. The addition of LcP to the PEDOT:PSS aqueous solution further promotes phase separation and lowers the temperature at which phase separation occurs, making it challenging to selectively crosslink the gel structure via photolithography. To clarify the solubility characteristics of PB‐CH, we varied the soft‐bake temperature. Notably, when dried at or below 60 °C, the PEDOT:PSS content falls below 50%, and the material does not fully dissolve but instead swells, retaining its solubility in water (**Figure** **3**a). At higher PEDOT:PSS content (>30%), films obtained from drying the PEDOT:PSS/LcP solution at 60 °C begin to exhibit insolubility in water due to phase separation. Within the range of PEDOT:PSS content values from 10% to 30%, the films dried at temperatures exceeding 80 °C also begin to exhibit insolubility in water. When the PEDOT:PSS content is reduced (<10%), films dried at temperatures below 100 °C exhibit good solubility in water. To further quantify the data, we investigate the impact of I2959 on the patterning and water content of direct photolithography PEDOT:PSS/LcP hydrogels during soft‐baking at 50 °C (Figure 3b). When the PEDOT:PSS content exceeds 40%, regardless of the I2959 concentration, the material dissolves in deionized (DI) water. In the range of 0% to 40% PEDOT:PSS solid content and 1%–7% I2959 content, patterned hydrogels are formed in DI water. However, when the PEDOT:PSS content is insufficiently low (<10%), the resulting hydrogel patterns gradually transition to white. The interactions between PEDOT:PSS and LcP further modify the gel particles of PEDOT:PSS, and the size of these particles may influence the resolution of direct photolithography. To explore the effect of PEDOT:PSS content on the particle size of PEDOT:PSS/LcP gel and subsequently optimize PEDOT:PSS/LcP for enhanced resolution, we examined the particle size at different PEDOT:PSS concentrations. It is worth noting that when the solid content of PEDOT:PSS is 30%, the measurements show that it has the smallest gel particle size^[^ ^41^ ^]^ (Figure S9, Supporting Information). At a PEDOT:PSS content of 30%, the prepared solution uniformly forms a film after spin‐coating, exhibiting consistent UV transmittance across different locations on the film. Additionally, the varying concentrations of I2959 exert minimal impact on the water content of the PB‐CH, which remains ≈71% (Figure 3c).

We selected PET and 3 m 300LSE as flexible substrates and used PDMS as a control due to its typically low bonding properties with PEDOT:PSS. A significant modulus difference is observed on the interfacial fit between cured PB‐CH and PDMS, while the Young's modulus gradient between PB‐CH and both PET and 3 m 300LSE varies more gently, indicating better interfacial compatibility (Figure 3d).^[^ ^42^ ^]^ The scratch test involves drawing a rigid stylus across the surface of the coating while continuously or gradually increasing the applied force in the direction of the coating thickness.^[^ ^43^ ^]^ The applied force and post‐scratch morphology are recorded to determine the critical force at which coating failure occurs (Figure 3e). This critical scratch force provides quantitative data for evaluating the adhesion of the PB‐CH film to the substrate. By comparing the failure forces, the adhesion strength of PB‐CH and PEDOT:PSS films can be assessed. Microscopic examination of the scratch test's surface morphology reveals that the PB‐CH film displays sharp, continuous scratches indicative of strong adhesion, while the PEDOT:PSS film detaches in larger pieces without clear scratch marks (Figure 3f). This observation suggests that PB‐CH exhibits significantly better adhesion to the substrate compared to PEDOT:PSS.

### 2.4 Photopatternability of PB‐CH

**Figure 4:**
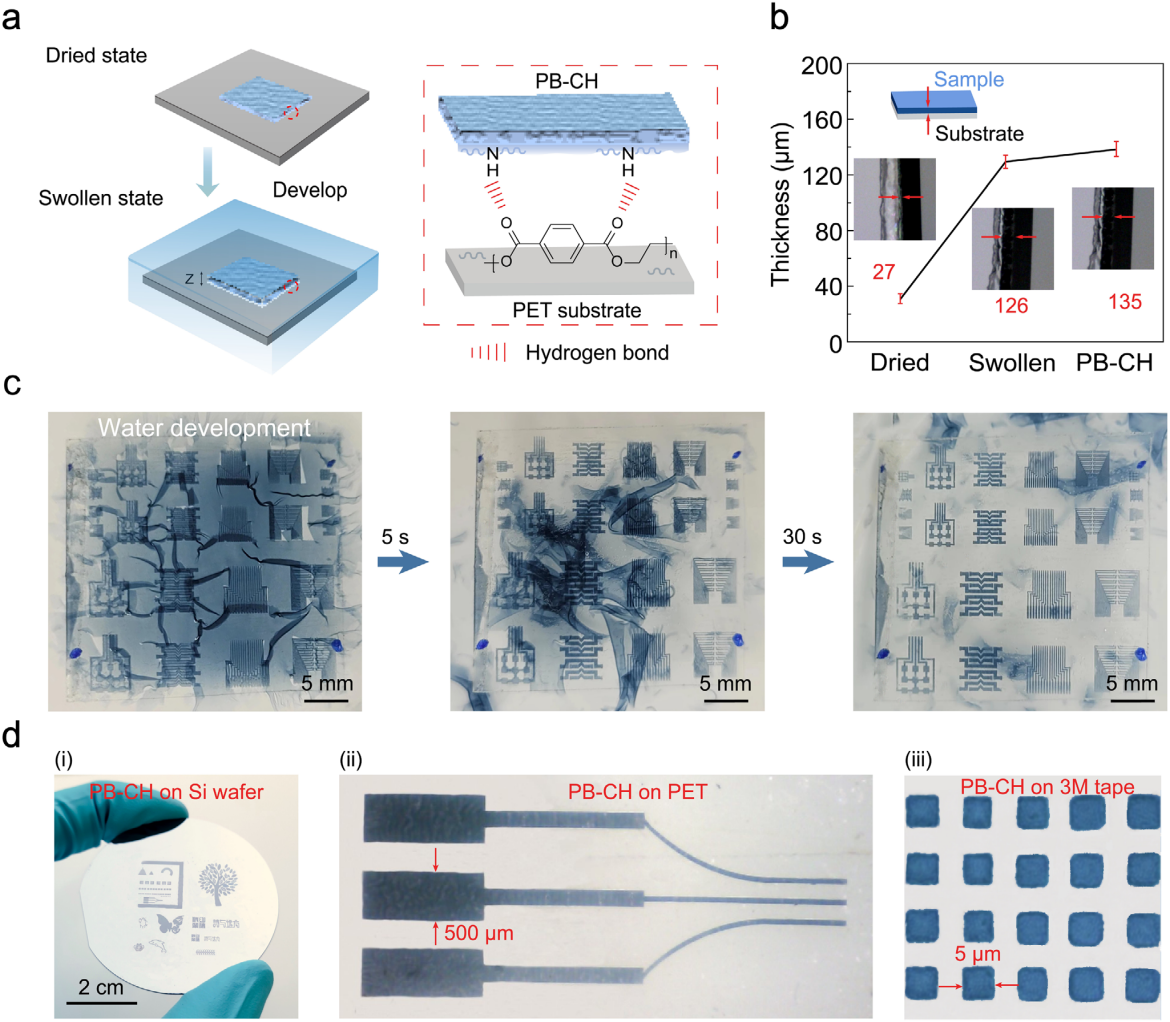
Anti‐swelling behaviors and demonstration of direct photolithography and high‐resolution PB‐CH on flexible substrate. a) Schematic illustration of anti‐swelling of UV‐cured PB‐CH film into hydrogel on flexible substrate. b) The thicknesses of dried PEDOT:PSS/LcP, swollen PEDOT:PSS/LcP, and PB‐CH: the average values are 27, 126, and 135 µm, respectively. Inset: side view of the sample showing the thickness. c) Experimental images for the development of direct photolithographic patterned PB‐CH. d) Optical images of diverse multiscale shapes and high‐resolution pattern arrays of PB‐CH.

The swelling behavior of PB‐CH is significantly influenced by the interaction forces between the substrate and the material. To enhance the integration and patterning reliability of PB‐CH on flexible substrates, polyethylene terephthalate (PET) film was selected as the substrate due to its oxygen‐rich groups. These groups form hydrogen bonds with the hydrophilic components of PB‐CH, such as the amino (─NH_2_) groups from LcP,^[^ ^44^ ^]^ thereby enhancing interfacial interactions and increasing adhesion (**Figure** **4**a). Additionally, the PET substrate was plasma‐treated to improve its hydrophilicity (Figure S12, Supporting Information).^[^ ^45^ ^]^ A photolithographic hydrogel precursor solution was then applied to coat the PET surface, followed by drying at 50 °C to remove water. UV light at a wavelength of 365 nm was used to initiate polymerization, effectively patterning the PB‐CH thin film layer. While UV treatment induces a structural transformation in PEDOT, converting its benzene structure to a quinone structure (Figure S13, Supporting Information), which enhances its conductivity,^[^ ^46^ ^]^ the low concentration of PEDOT:PSS in PB‐CH results in suboptimal electrical properties. In the lithographic process, we used acetic acid (HOAc), which has been reported as a poor solvent for PEDOT:PSS and can induce the aggregation of PEDOT chain.^[^ ^18^ ^]^ Specifically, HOAc facilitates the vertical rearrangement of polymer chains, likely due to the interactions between the solvent and the PSS component of the PEDOT:PSS, further promoting aggregation of the PEDOT chain and phase separation. This behavior results in a slight increase in the ratio between PEDOT and PSS relative to the light‐curing polymer (LcP) following HOAc treatment. This effect is attributed to the interaction forces between the PSS and LcP networks, with only a small portion of the PSS washed away during the immersion process (Figures S14 and S15, Supporting Information). The film undergoes a considerable increase in thickness, from 27 ± 3.5 µm in the dry state to 126 ± 4.6 µm in the hydrogel state, representing a change of 366%. After treatment with HOAc and soaking in DI water to replace the HOAc, forming PB‐CH, the thickness increased to 135 ± 5.3 µm, showing only a slight change (Figure 4b).

Under optimal UV irradiation conditions, the aqueous stability of PB‐CH increases with higher UV doses (Figure S16, Supporting Information). A key advantage of PB‐CH fabricated via direct photolithography is its ability to swell into hydrogels in DI water, facilitated by a water‐based development process (Figure 4c; Movie S1, Supporting Information). To validate the resolution of PB‐CH, a variety of patterns were generated using direct photolithography (Figure 4d(i,ii); Figures S17–S19, Supporting Information). Additionally, high‐resolution patterns with a feature size of 5 µm were achieved using a corresponding high‐resolution photomask (Figure 4d(iii)). Notably, the patterned PB‐CH demonstrates long‐term stability in an aqueous environment, showing no apparent dissolution over time (Figure S20, Supporting Information).

### 2.5 Electrical, Mechanical, and Electrochemical Properties of PB‐CH

**Figure 5:**
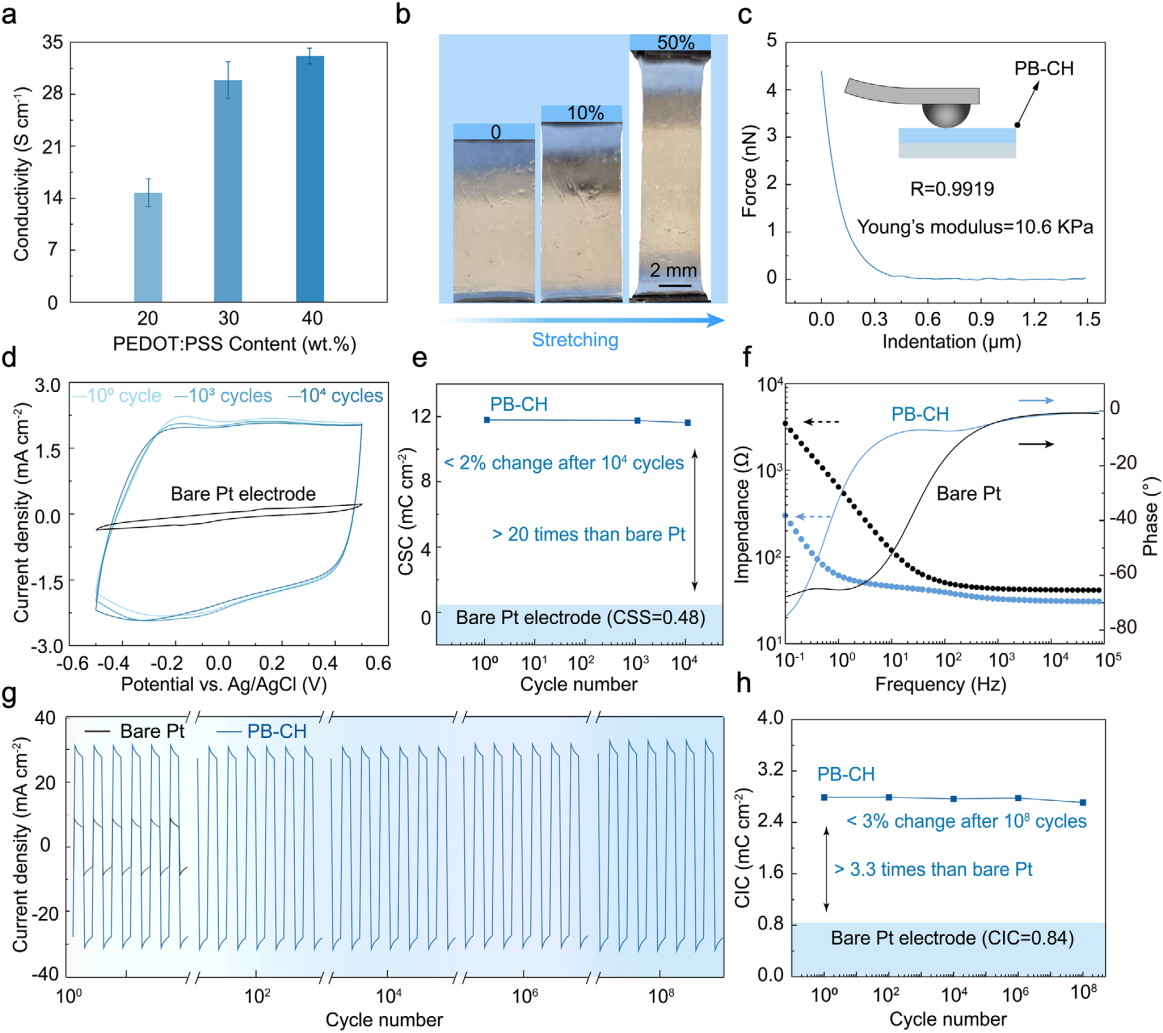
Mechanical and electrochemical properties of PB‐CH. a) Dependence of conductivity on the proportion of PEDOT:PSS. b) Optical image demonstrating the shape change of the PB‐CH pattern during stretching. c) Nanoindentation of PB‐CH with the contact point‐based model fit. d) CV characterization for a direct photolithographic PB‐CH on Pt electrode after 10 000 cycles. e) Top: The change in CSC of PB‐CH after 10 000 cycles. Bottom: The change in CSC of the bare Pt electrode after 10 000 cycles. f) Impedance spectra of a bare Pt electrode and the PB‐CH. The scatter plot illustrates the impedance magnitude, while the broken lines denote the phase angle. g) Current density versus time curves for a Pt electrode and the PB‐CH after 10 00 00 000 cycles. h) Top: The change in CSC of PB‐CH after 10 00 00 000 cycles. Bottom: The change in CIC of the bare Pt electrode.

The electrical, mechanical, and electrochemical stability of conducting hydrogels are critical factors in assessing functionality and reliability. In this study, LcP was blended with PEDOT:PSS solution at 60–80 mass % of the final hydrogel precursor solution and dried on a substrate. Following UV and HOAc treatments to enhance aqueous stability, the PB‐CH with biphasic structure achieved a conductivity of ≈30 S cm^−1^ with a PEDOT:PSS to LcP ratio of 30:70 (**Figure** **5**a). As the PEDOT:PSS content increases, the conductivity of PB‐CH improves. However, when the PEDOT:PSS content exceeds 30%, the conductivity reaches a plateau, and the mechanical properties of the PB‐CH significantly decrease (Table S1, Supporting Information). Dried PEDOT:PSS on low‐adhesion substrates (e.g., PDMS) may detach when swollen into a hydrogel. Since plasma treatment is not dependent on the specific characteristics of the substrate, we selected 3 m 300LSE as the soft substrate. After direct photolithography, a fully photopatterned rectangular structure of PB‐CH was formed on the 3 m 300LSE substrate. This pattern remained intact and free of cracks when stretched to 50% strain without visible cracks, indicating robust mechanical integrity (Figure 5b; Figure S21, Supporting Information). Notably, in order to quantify the softness of the PB‐CH, we performed nanoindentation testing (Figure 5c). The hydrogel exhibited a Young's modulus of 10.6 kPa, a value closer to that of human tissue compared to soft elastomers such as PDMS, which typically possess a Young's modulus ranging from 1 to 10 MPa.^[^ ^47^ ^]^ More importantly, PB‐CH demonstrated superior performance compared to state‐of‐the‐art patterned PEDOT:PSS hydrogels (Table S2, Supporting Information), highlighting its effectiveness and potential for high‐performance flexible electronics.

The key to ensuring long‐term operation lies in maintaining electrochemical stability in physiological or buffered aqueous environments. The long‐term electrochemical stability of PB‐CH was evaluated in phosphate‐buffered saline (PBS). The charge storage capacity (CSC), a critical performance metric for bioelectronic electrodes, was compared with that of platinum (Pt), a conventional metal electrode material known for its electrochemical stability. PB‐CH demonstrated outstanding stability over 10 000 charge–discharge cycles, with only minimal changes observed in the cyclic voltammetry (CV) curves (Figure 5d). Notably, the CSC of PB‐CH is over 20 times higher than that of Pt electrodes, showing its superior energy storage capability. While the CSC of PB‐CH exhibited only a 2% decrease after 10 000 scanning cycles, Pt electrodes demonstrated significantly lower charge storage capacity, highlighting the exceptional stability and durability of PB‐CH under long‐term cycling conditions (Figure 5e). Owing to the porous network structure, PEDOT‐based hydrogels allow electrolyte ions to penetrate deeply into their interior, unlike metallic electrodes, where ion interactions are predominantly limited to the surface. This structural advantage substantially reduces overall impedance.^[^ ^6^, ^29^ ^]^ The electrochemical impedance spectroscopy (EIS) was measured to evaluate the areal impedance of PB‐CH in comparison to the bare Pt electrode. Areal impedance is defined as the impedance normalized by the electrode surface area, allowing for a direct comparison between different electrode materials. As shown in Figure 5f, PB‐CH exhibited significantly lower impedance compared to the Pt electrode across all frequency ranges (10^−1^–10⁵ Hz), with a particularly pronounced reduction at low frequencies. Moreover, the PB‐CH displayed a phase angle closer to 0° across the 1–100 Hz frequency range, indicating that impedance was primarily dominated by resistance.^[^ ^3^, ^48^ ^]^ This behavior is critical for bioelectronic applications as it minimizes phase lag in signal transmission and ensures efficient charge transfer, preserving the temporal accuracy of bioelectronic signals. To evaluate its potential for bioelectronic applications, the charge injection capacity (CIC) of PB‐CH was assessed in the context of electrical stimulation. PB‐CH achieved a CIC of ≈2.7 mC cm^−^^2^, over three times higher than that of Pt electrodes (0.84 mC cm⁻^2^), demonstrating that PB‐CH's electrical performance remains uncompromised (Figure 5g). Furthermore, after 100 million cycles of biphasic charge injections, the CIC of PB‐CH decreased by only ≈3%, indicating remarkable long‐term stability (Figure 5h).

## 3 Conclusion

In summary, we successfully synthesized a photosensitive biphasic conducting polymer hydrogel (PB‐CH) by combining PEDOT:PSS as the conductive phase and a light‐curable polymer matrix as the mechanical phase. This innovative composition enabled the creation of a hydrogel with excellent electrical conductivity of 30 S cm^−1^, a tensile strain of 50%, and robust electrochemical stability. We developed a direct photolithography process optimized for PB‐CH, enabling high‐resolution patterning at the 5 µm scale directly on flexible substrates. This approach eliminates the need for complex transfer processes and reduces fabrication steps, offering a streamlined and scalable solution for flexible electronics. Through a systematic examination of the photolithography parameters and curing mechanism, we demonstrated that PB‐CH can be reliably patterned into various electrode structures, expanding the range of fabrication methods for flexible and bioelectronic devices. Our work presents a promising strategy for the high‐resolution patterning of high‐performance conducting polymer hydrogels, paving the way for innovative applications in advanced flexible electronics and bioelectronics.

## 4 Experimental Section

### Materials

The PEDOT:PSS aqueous solution (1.3 wt.% solid content, CLEVIOS PH1000) was purchased from Heraeus and used as received. Polytetrahydrofuran (PTMG1000), dicyclohexylmethane diisocyanate (HMDI), hydroxyethyl acrylate (HEA), 2,2‐bis(hydroxymethyl)propionic acid (DMPA), dibutyltin dilaurate (DBTDL), acrylic acid (AA), acrylamide (AM), 2‐hydroxy‐4′‐(2‐hydroxyethoxy)‐2‐methylpropiophenone (Irgacure 2959), and pure acetic acid (HOAc) were purchased from Aladdin and used without further purification. BY3303 was purchased from Beijing Baiyuan Chemical Co., Ltd., and Capstone FS‐30 was purchased from DuPont and used as received. The deionized (DI) water (>18.25 MΩ cm) used in all experiments was purified by a Purifier FST‐I‐B10 (Shanghai Fu Shi Instrument Equipment Co., Itd). The substrates used in photolithography were Si wafer (0.5 mm thickness, 50.8 mm in diameter), 3 m 300LSE with a thickness of 0.17 mm, PDMS with a thickness of 0.05 mm, and PET with a thickness of 0.3 mm were purchased from Shenzhen Weina Electronie Technology Co. Ltd, 3M China Ltd., and Zhongshan Hongyi Film Technology Co. Ltd, respectively.

### Synthesis of LcP

The synthetic route of light‐curing polymer (LcP) is illustrated in Figure S1 (Supporting Information). In a 250 mL round‐bottom flask, 27.31 g HMDI, 52.11 g PTMG1000, 20.08 g DMPA, and 2.33 g BY‐3303 were added sequentially and stirred to form a uniform dispersion. The resulting dispersion was then switched to the flask, along with a magnetic stirrer. The mixture was degassed under a flow of nitrogen for 30 min, then 1 g of DBTDL was added, and the flask was sealed. The flask was placed in an 80 °C oil bath and stirred at 600 rpm for 2 h. Next, 10.14 g of HEA was added to the reaction system, and the reaction continued at 80 °C until the characteristic absorption peak of the −NCO ≈2270 cm^−1^ disappeared, as confirmed by infrared testing of the samples. After the reaction, the mixture was cooled to room temperature and purified repeatedly using a mixture of water and methanol.

### Preparation of the Photosensitive Biphasic PB‐CH Solution

A 1.3 wt.% aqueous solution of PEDOT:PSS (PH1000) was mixed with LcP to achieve PEDOT:PSS weight ratios ranging from 0:1 to 0.5:1. After stirring for 8 h at room temperature, 1–5 wt.% of Irgacure 2959 (relative to LcP) and 1 wt.% FS‐30 (relative to PH1000) were added to the final ink, which was then stored under light‐shielded conditions. Subsequently, the precursor was coated onto substrates such as silicon (Si) wafer, PDMS, PET, and 3 m 300LSE using solution processes like spin coating. Finally, the coated substrates were dried at 50 °C for 8 h in the dark.

### Direct Photolithography of PB‐CH

The direct lithography system consisted of a 365 nm UV wavelength source and a water‐ and oxygen‐free glove box, which was used to pattern the precursor of PB‐CH with photomasks. The optimal parameters in this study were identified as 75 mW cm^−2^ for the PEDOT:PSS concentration of 30% in the precursor solution. After UV curing, the sample was immersed in DI water to capitalize on the differences in solubility between the cured and uncured regions. The PB‐CH pattern was examined using the 5MPAM73915 Series (Dino‐Lite) and DV500 (Chongqing Optec Instrument Co. Ltd.) and compared with the pattern on the photomask on the UV‐transmitting photomask.

### Density Functional Theory (DFT) Simulation

PEDOT and PSS were initially generated using the genmer module within the Molclus software, resulting in 100 randomized structures (traj.xyz). Subsequently, optimization calculations were conducted using the XTB software under the GFN2‐xtb method. The optimized energies and coordinates (isomers.xyz) were recorded, and the structures were then categorized into groups based on their energy similarity. These structures were further sorted in ascending order of energy and saved in cluster.xyz. From cluster.xyz structures with the lowest energies were selected as the foundation for constructing PEDOT:PSS/LcP, named PB‐CH. Subsequently, the operations of PEDOT and PSS were used, and both operations needed to take into account the solvation effect with the GBSA implicit solvent model.

The SMD implicit solvation model was used to account for the solvation effect. The binding energies of both PEDOT:PSS and PEDOT:PSS/LcP compounds were calculated according to the following equations:

(1) $$E_{\mathrm{bind}}=E_{\mathrm{comp}\mathrm{lex}}-\left(E_{\mathrm{partA}}+E_{\mathrm{partB}}\right)$$

All calculations were carried out by the latest version of ORCA quantum chemistry software (Version 5.0.4).

For geometry optimization calculations, the corrected version of the r2SCAN exchange‐correlation function proposed by Grimme (so‐called r2SCAN‐3c) was adopted.

### Nanoindentation Characterizations

Nanoindentation characterizations of PB‐CH were performed using an atomic force microscopy (AFM) facility (Dimension XR, Bruker) in a wet environment. A spherical tip with a 6 µm radius (MLCT‐O10, F triangle, Bruker) was used for the nanoindentation measurements. All nanoindentation images were recorded at mean indentation tip velocity of 2752 nm s^−1^ and mean indentation loading rate of 66.8 nN s^−1^. Young's moduli of the hydrogel were obtained by fitting force versus indentation curve with a contact point‐based model fit. For nanomechanical imaging, AFM measurements of nanomechanical imaging were taken using a Bruker Dimension Icon instrument and an RTESPA‐300 atomic force microscope probe (from Bruker, a tip radius of 8 nm). All nanomechanical images were taken at a scanning frequency of 0.5 kHz. The films were prepared by dropping a PB‐CH precursor solution onto a plasma‐treated flexible substrate, vacuum‐drying at 50 °C in the dark, and then cutting the cross‐section with a razor blade (after exposure).

### Scratching Test

The PEDOT:PSS solution and the precursor (with a weight ratio of 0.3:1 PEDOT:PSS to PEDOT:PSS/LcP) were coated on PET substrates, followed by a soft‐bake at 50 °C. The scratch test was conducted on the samples after the soft‐bake using Anton Paar UNHT equipped with a standard tip (curvature radius of 100 µm). The test protocol included maintaining an initial load of 0.03 N, with a loading rate of 5 N min^−1^ until reaching a peak load of 5 N, followed by unloading. The scratching speed was set at 3 mm min^−1^. Furthermore, scratch images were captured using an in situ scanning probe microscope.

### Electrical Characterization

The electrical conductivity of the PB‐CH was measured using a four‐point probe (Keithley 2700 digital multimeter) system. The samples were cut into rectangular shapes (2 mm × 10 mm). Platinum (Pt) wire electrodes (0.5 mm in diameter) were attached to both ends of the samples using conductive silver paste, which served to minimize interfacial resistance between the electrodes and the PB‐CH surface. This approach ensured accurate conductivity measurements by reducing contact resistance effects. Furthermore, a humidifier was used throughout the experiment to maintain sufficient moisture content in the samples, preventing dehydration. At least three different samples were used for each test. The electrical conductivity (σ) is calculated according to Equation (2):

(2) $$\sigma =\frac{l}{R\;w\;t}=\frac{I\times L}{V\times w\times t}$$

where *w* is the width of the sample, *L* is the distance between the two electrodes for voltage measurement, *I* is the current flowing, *V* is the voltage across the electrodes, *t* is the thickness of the sample, and *R* is the resistance of the samples measured using the direct current.

Cyclic voltammetry (CV), potentiostatic electrochemical impedance spectroscopy (EIS), and charge injection capacity (CIC) of the PB‐CH modified Pt substrates were performed by using an electrochemical potentiostat/galvanostat (Interface 1010–29074; Gamry Instruments) with a standard three‐electrode electrolytic cell where the sample served as the working electrode, the platinum sheet acted as the counter electrode, the Ag/AgCl line functioned as the reference electrode, and phosphate‐buffered saline (PBS) solution was employed as the electrolyte. The scanning rate was set as 150 mV s^−1^, and the scanning potential range was set as −0.5 V ≈ 0.5 V. All the samples were immersed in PBS solution for 6 h before measurement.

The CSC values of the CV curves are calculated using Equation (3):

(3) $$\mathrm{CSC}=\int_{E_{i}}^{E_{t}}\frac{i(E)}{2\mathrm{vA}}\;\mathrm{dE}\;$$

where E_i_ and E_t_ are the upper and lower limits of the potential, *v* is the potential scanning rate, i is the current at each potential, and A is the area of the PB‐CH modified on the Pt electrodes, respectively.

The variation in CSC is computed from Equation (4):

(4) $$\Delta \mathrm{CSC}=\frac{\mathrm{CS}C_{f}-\mathrm{CS}C_{i}}{\mathrm{CS}C_{i}}\;\cdot \;100\%$$

where CSC_f_ is the charge storage capability after treatment, such as 10 000 cycles CV scanning, while CSC_i_ is the charge storage capability of the as‐prepared PB‐CH.

EIS measurements of the PB‐CH on the Pt electrode were obtained with a sine wave (10^−1^–10^5^ Hz) and a signal amplitude of 10 mV to evaluate the electrical impedance and their stability. Both the phase angle data and impedance were recorded across the entire frequency range.

For CIC of the PB‐CH‐modified electrode, the electrochemical current pulses ranging from −0.5 to 0.5 V versus Ag/AgCl were applied to demonstrate the excellent stability of the hydrogel electrode after 10 000 cycles. CIC is calculated using the following Equation (5):

(5) $$\mathrm{CIC}=\frac{Q_{\mathrm{inj}\left(c\right)}}{A}+\frac{Q_{\mathrm{inj}\left(a\right)}}{A}$$

where CIC represents charge injection capacity of PB‐CH. Qinj(c) denotes the total injected charge during the cathodal phase following the reduction potential, while Qinj(a) denotes the total injected charge during the anodal phase following the oxidation potential, and A represents the area of the hydrogel, respectively.

The variation in CIC is computed from Equation (6):

(6) $$\Delta \mathrm{CIC}=\frac{\mathrm{CI}C_{f}-\mathrm{CI}C_{i}}{\mathrm{CI}C_{i}}\;\cdot \;100\%$$

where CIC_f_ is the charge injection capacity after treatment, such as 10 00 00 000 cycles, while CIC_i_ is the charge injection capacity of the as‐prepared PB‐CH.

### Water Content Test

The sample was immersed in DI for 24 h to achieve hydration. Subsequently, the surface water was wiped off before testing. The weight of the hydrated sample was measured using an electronic balance and denoted as W_h_. After weighing, the sample was dried at 60 °C for 24 h. The dried sample was then obtained, and its weight was recorded as W_f_. At least three different samples were used for each test. The water content of the hydrogels is calculated according to Equation (7):

(7) $$\mathrm{Water}\;\mathrm{content}=\frac{W_{h}-W_{f}}{W_{h}}\;\cdot \;100\%$$

### Mechanical Characterization

All samples for mechanical characterizations were prepared on a flexible 3 m VHB substrate. The stretchability of the hydrogels was measured by a mechanical testing machine (Instron 68TM‐5) equipped with a 100 N load‐cell. Additionally, a humidifier was employed to maintain adequate moisture levels in the samples throughout the experiment, preventing dehydration.

### Photograph and Video Recording

Unless stated otherwise, all photographs were captured using 5MPAM73915 Series (Dino‐Lite) and DV500 (Chongqing Optec Instrument Co. Ltd.), and the videos were taken with Galaxy S24.

## Conflict of Interest

The authors declare that they have no conflicts of interest.

## Author Contributions

W.W., J.L., and H.L. contributed equally to this work as co‐first authors. B.L. conceived the idea and directed the project. W.W. and B.L. designed the study. W.W. and J.L. conducted the experiments. W.W., J.L., H.L., and B.L. analyzed and interpreted the results. All the authors contributed to the writing and editing of the paper.

## Supporting information



Supporting Information

Supplemental Movie 1

## Data Availability

The data that support the findings of this study are available from the corresponding author upon reasonable request.
