# Supplementary material for: Photopatternable PEDOT:PSS Hydrogels for High‐Resolution Photolithography
Source: Adv Sci (Weinh). 2025 Mar 24;12(19):2414834. doi: 10.1002/advs.202414834 (PMC12097077; doi:10.1002/advs.202414834)
Supplement: Supplementary file 1 — Supporting Information [file ADVS-12-2414834-s001.docx]

**Supporting** **Information**

Photopatternable PEDOT:PSS hydrogels for high-resolution photolithography

Wen Wang ^a^ **^†^**, Jingcheng Liu ^b^ **^†^**, Hai Li ^a^ **^†^**, Yi Zhao ^c^, Qiaobo Wang ^a^, Rongtai Wan ^a^, Jingkun Xu ^d^, and Baoyang Lu ^a^ *

^a^ Jiangxi Provincial Key Laboratory of Flexible Electronics, Flexible Electronics Innovation Institute, Jiangxi Science & Technology Normal University, Nanchang 330013, P. R. China

^b^ School of Chemical and Material Engineering, Jiangnan University, Wuxi 214122, P. R. China

^c^ Robotics Institute and State Key Laboratory of Mechanical System and Vibration, School of Mechanical Engineering, Shanghai Jiao Tong University, Shanghai 200240, P. R. China

^d^ School of Water Resources & Environmental Engineering, East China University of Technology, Nanchang 330013, PR China

^*^ **Corresponding author:** Baoyang Lu, luby@jxstnu.edu.cn.

^†^ These authors contributed equally to this work.


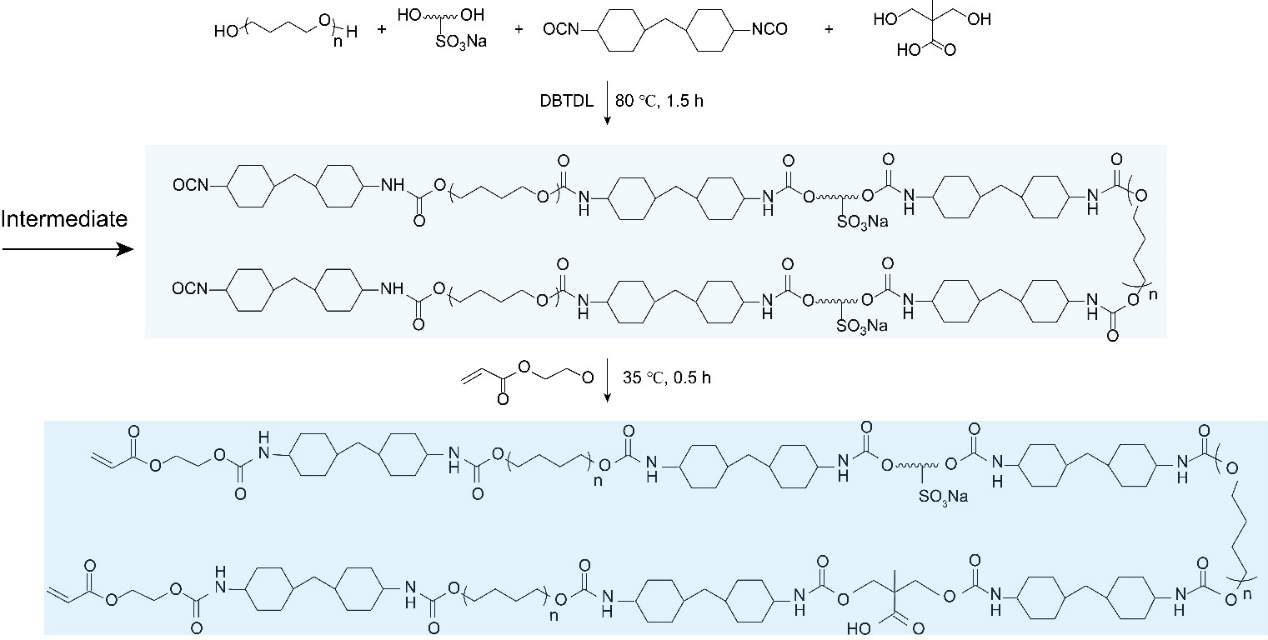


**Figure S1.** Synthesis of LcP photocurable polymer. The final LcP consists of a polytetrahydrofuran (PTMG1000), a main chain with sulfonic acid group (BY3303), and hydroxyethyl acrylate(HEA) terminal.

1H NMR (400 MHz, CDCl3, δ) 6.38 (ddd, J=17.3, 4.9, 1.4 Hz, 1H), 6.09 (ddd, J=16.9, 10.4, 6.3 Hz, 1H), 5.80 (ddd, J=10.4, 4.4, 1.5 Hz, 1H), 4.47–4.41 (m, 1H), 4.31–4.21 (m, 3H), 3.98 (s, 4H), 3.84–3.77 (m, 2H), 3.72–3.65 (m, 2H), 3.34 (tq, J=4.7, 2.5 Hz, 32H), 2.32–2.19 (m, 3H), 1.92 (d, J=11.9 Hz, 2H), 1.59 (s, 9H), 1.62–1.55 (m, 1H), 1.58–1.48 (m, 21H), 1.50 (s, 6H), 1.47 (s, 1H), 1.40–1.29 (m, 2H), 1.19 (s, 3H), 1.11–0.95 (m, 3H), 0.90 (qd, J=6.8, 2.9 Hz, 4H), -0.07 (s, 1H), (Fig. S2). Mw = 47,494 g mol–1, PDI = 1.07, (Fig. S4).


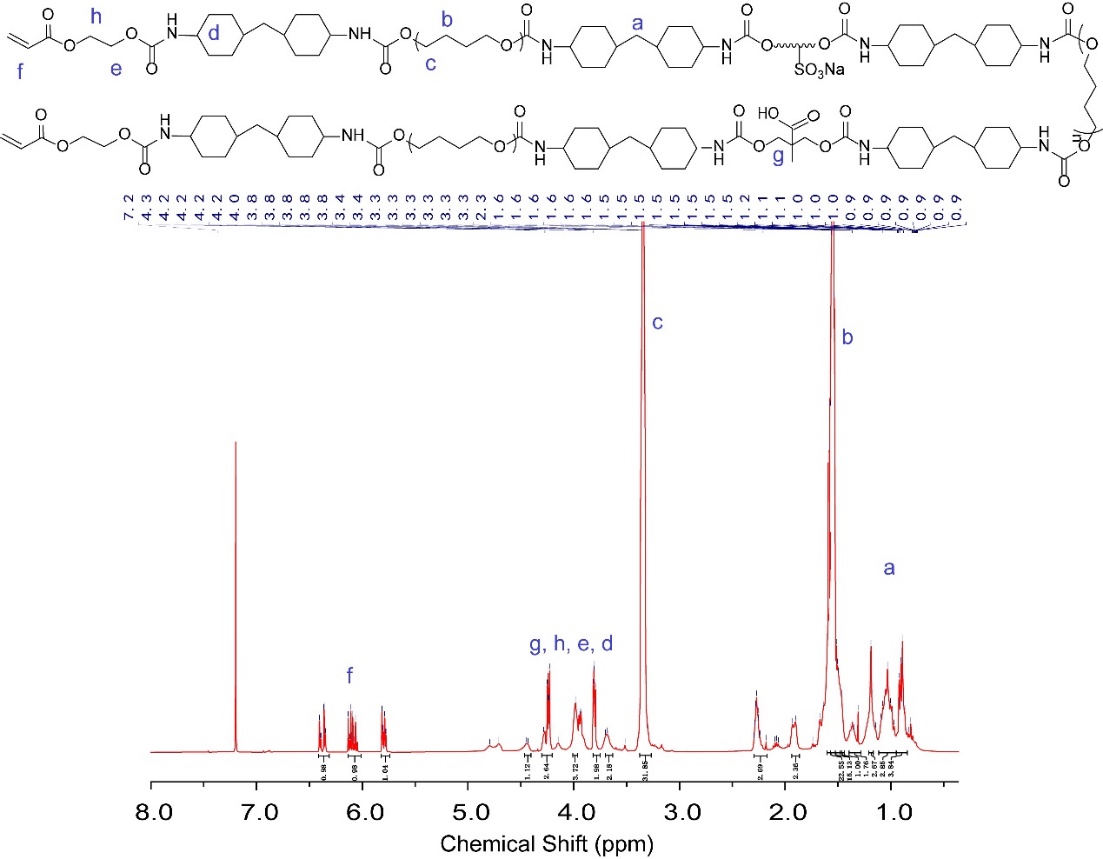


**Figure S2.** NMR spectrum of LcP in CDCl_3_.


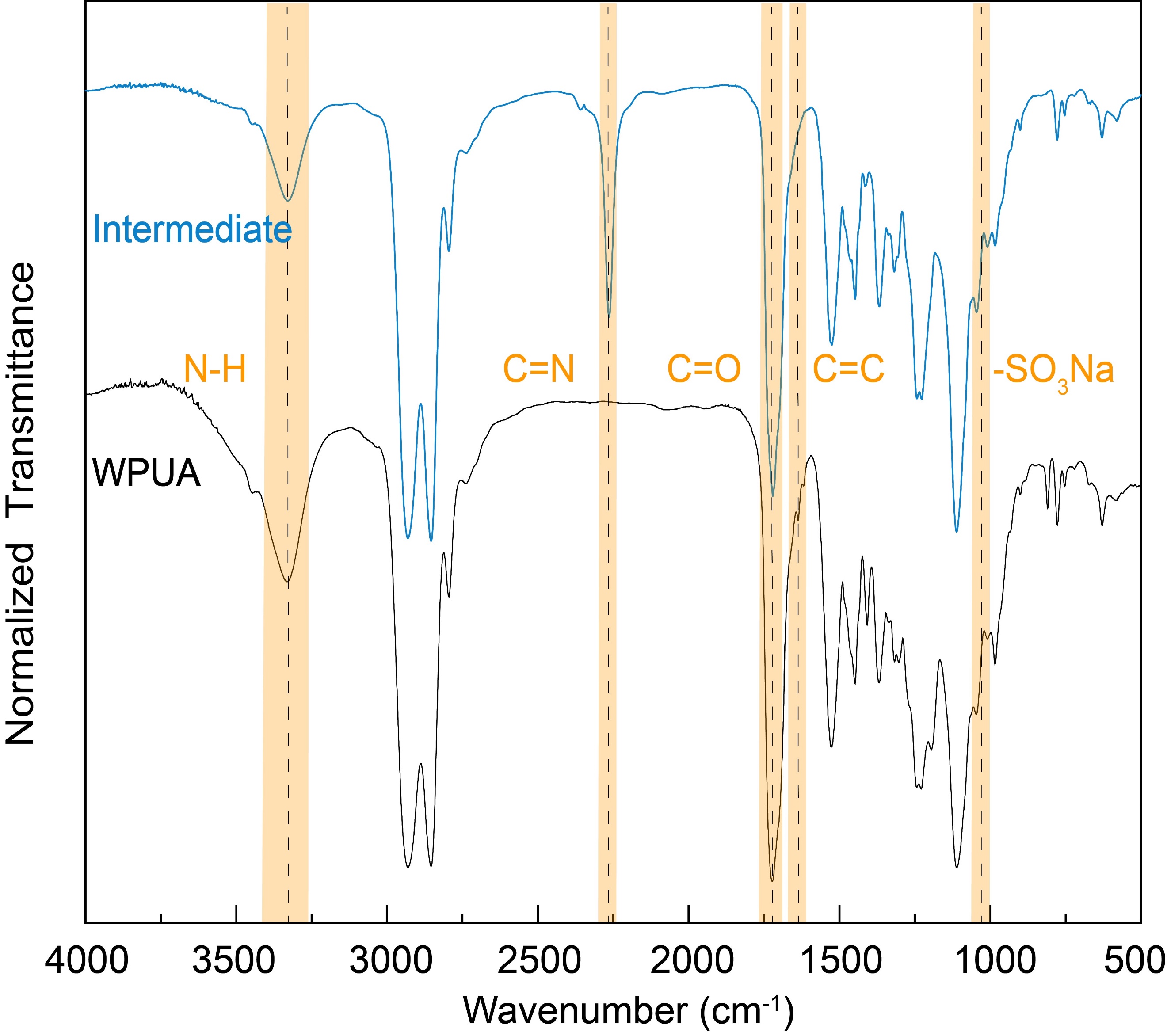


**Figure S3.** FT−IR spectra of LcP and intermediate. e prominent distinction lies at 2625 cm^−1^ and 1630 cm^−1^, indicating the transition from the intermediate state to the final LcP. This transition signifies the disappearance of the −C=N− at 2625 cm^−1^ and the formation of the −C=C− at 1630 cm^−1^. It can be inferred that the utilization of HEA as a terminal group leads to the formation of the polymerizable LcP.


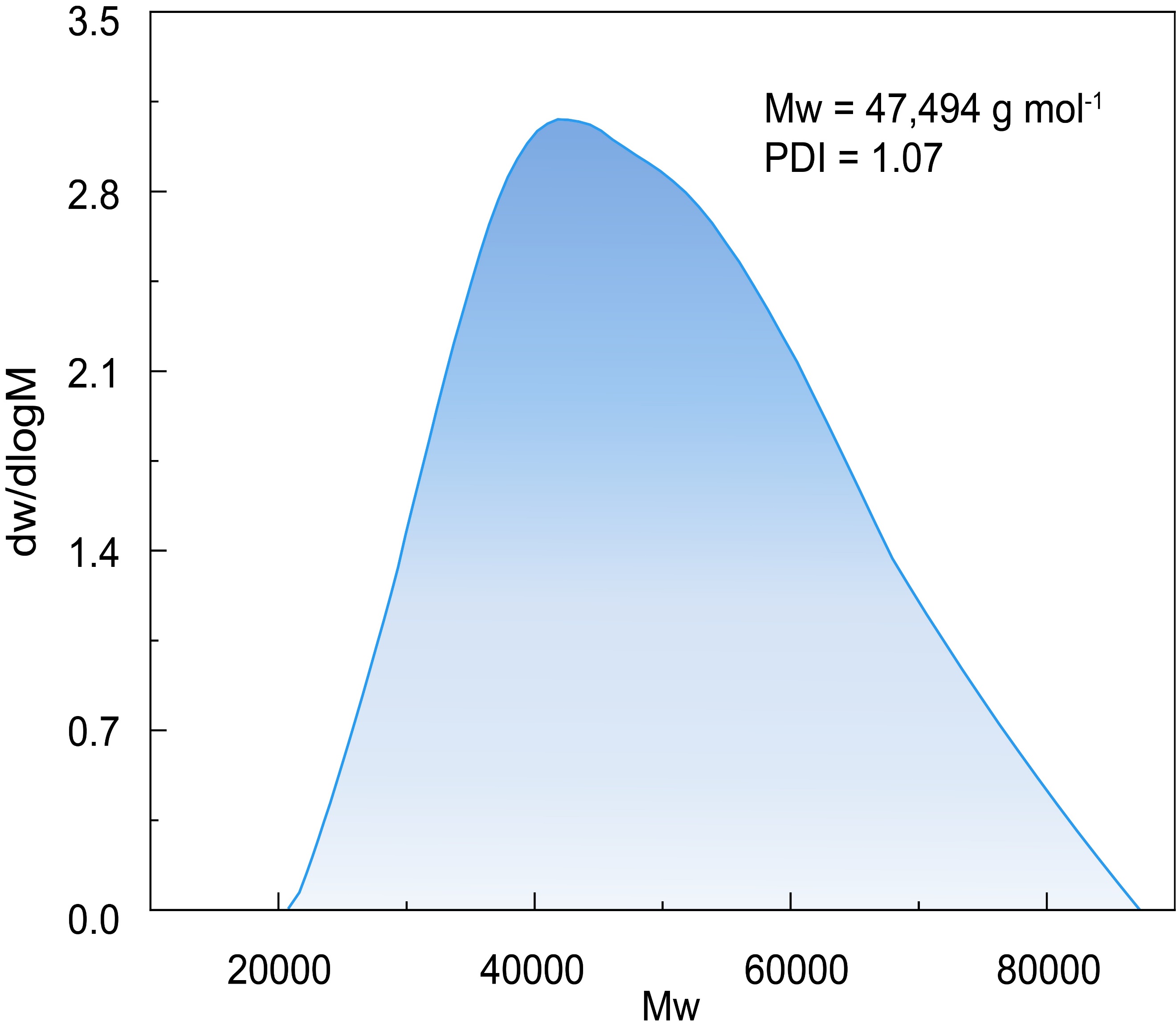


**Figure S4.** Overlaid Gel permeation chromatography (GPC) traces of LcP with water as the eluent.

**S.II. Instrumentation and characterizations.**

Atomic force microscopy (AFM) images were collected in contact mode using a Bruker dimension XR and Bruker Dimension Icon . X-ray photoelectron spectroscopy (XPS) was performed on a Thermo Scientific K-Alpha. Gel permeation chromatography (GPC) was conducted using an Agilent GPC 50 at 298 K with concentration of 0.10 mg/ml and water as the eluent. Nuclear magneticresonance (NMR) spectroscopy was performed on a BRUKER 400 UltraShield^TM^. Fourier-transform infrared (FTIR) spectrometer was performed on a Perkin-Elmer Spectrum Two, covering the wavelength range of 4000−500 cm^−1^, with a spectroscopy resolution of approximately 4 cm^−1^. Raman spectra was obtained using a Horiba LabRAM HR Evolution in the wavelength range of 50−4000 cm^−1^ with an excitation wavelength of 532 nm Ultraviolet-Visible (UV-Vis) spectroscopy was performed on a Hitachi UH4150, covering the wavelength range of 1000−250 cm^−1^, with a spectroscopy resolution of approximately 4 cm^−1^ Dynamic light scattering (DLS) measurements were conducted using the Particle Sizing Systems Nicomp N3000 instrument with a HeNe laser (514.4 nm) serving as the light source. PB-CH thickness values were measured using a Dino−Lite 5MPAM73915 Series. Rheological characterization was conducted using a TA Instruments AR−G2 equipped with 20 mm diameter steel parallel−plate geometry, covering logarithmically spaced shear rates ranging from 0.01 s^−1^ to 1,000 s^−1^. The scratch test was conducted using the Anton Paar UNHT instrument.


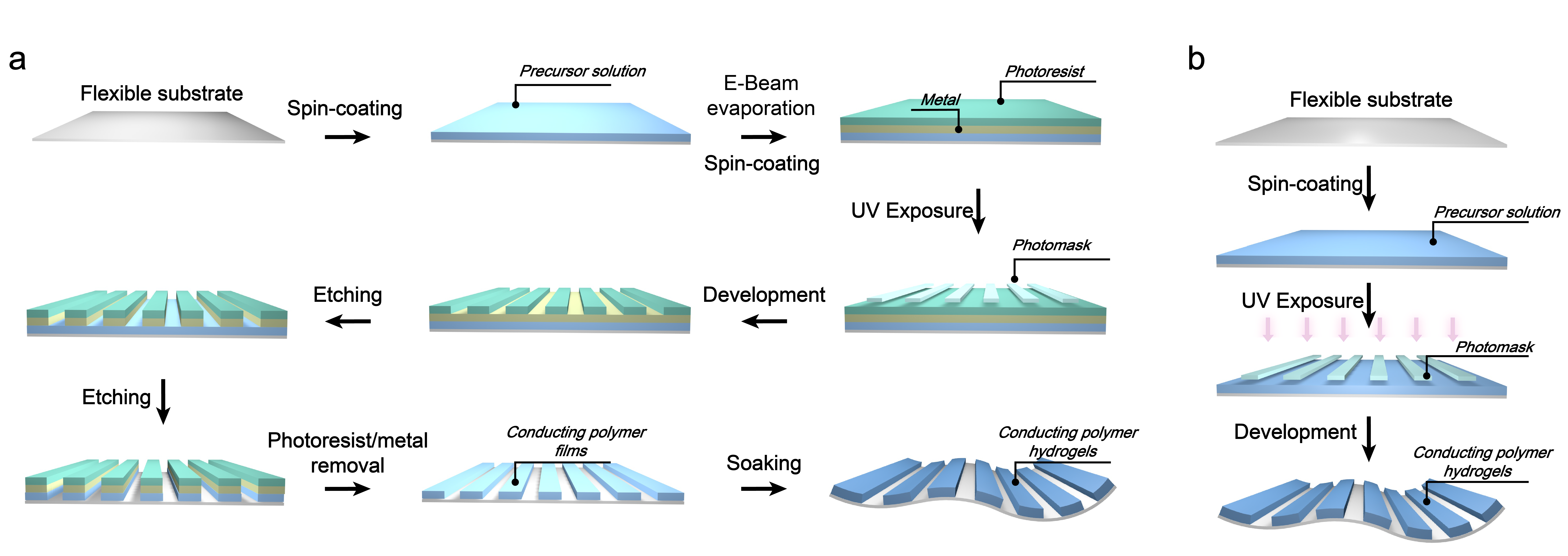


**Figure S5.** Comparison of different photolithography processes. (a) Conventional photolithography processes and (b) Direct photolithography based on PEDOT:PSS-based conducting polymer hydrogel. The conventional photolithography involves complex and time-consuming multistep process. In the process, the Direct photolithography are three-step without organic solvent.


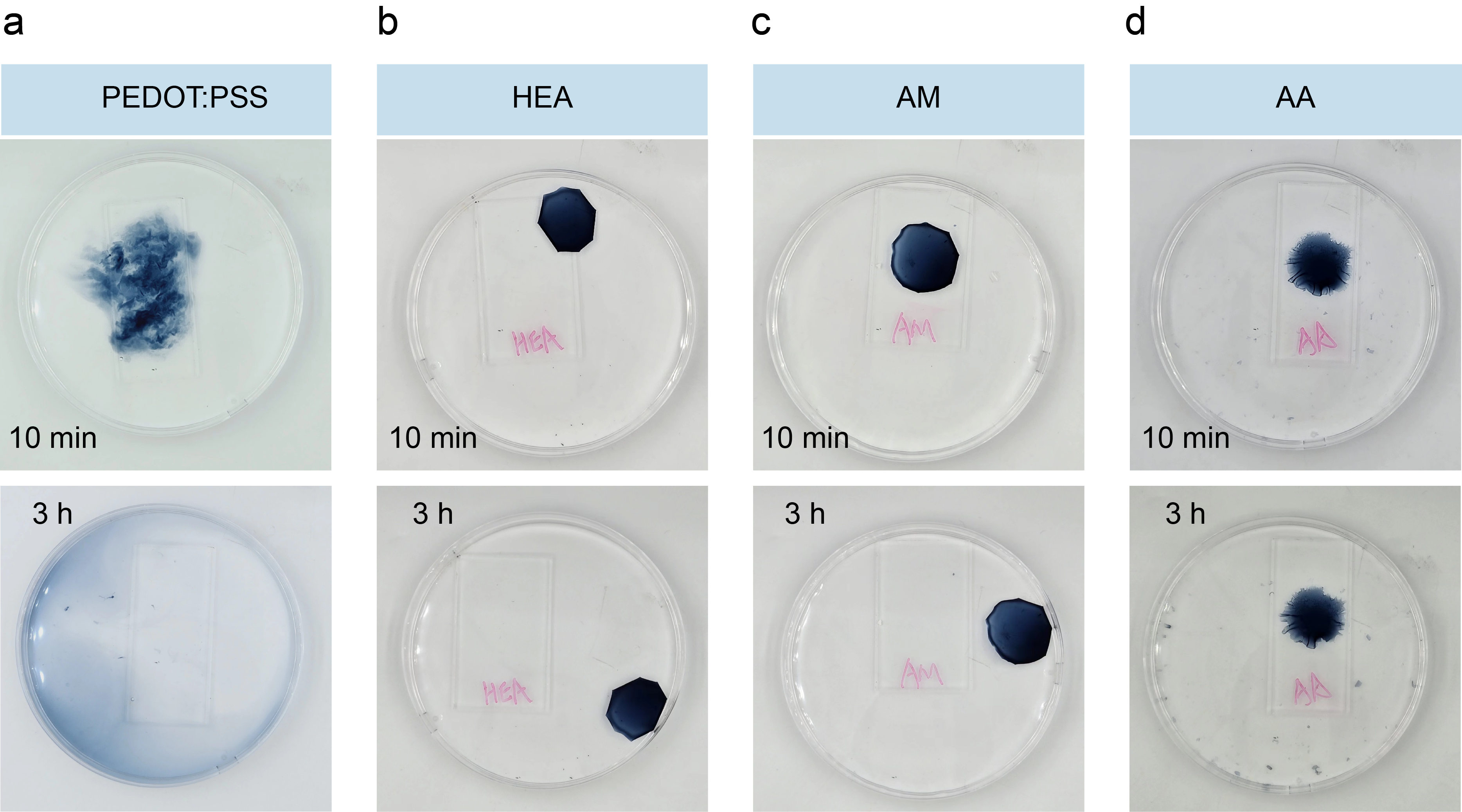


**Figure S6.** Dissociation of conducting polymer hydrogels. (a) The pristine PH1000, upon drying at 50 °C, swells and readily dissociates into fragmented microgels rather than forming a stable hydrogel. (b), (c), and (d), Swelling of monomers(HEA, b) (AM, c) (AA, d) separately added to PH1000 without chemical crosslinking and initiator, dried at 50 ℃, and swelled in water to form hydrogels.


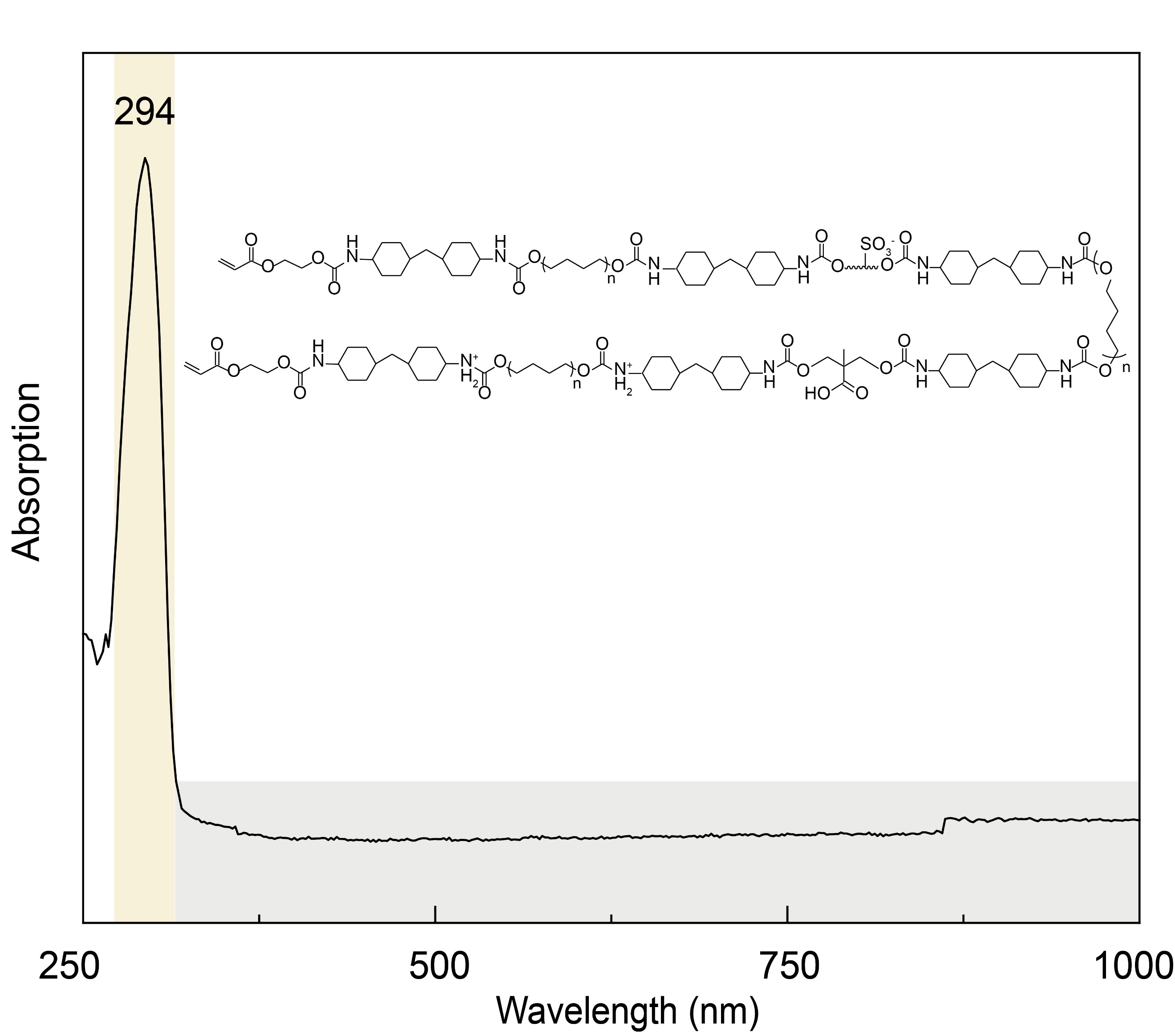


**Figure S7.** UV-vis spectra of LcP.


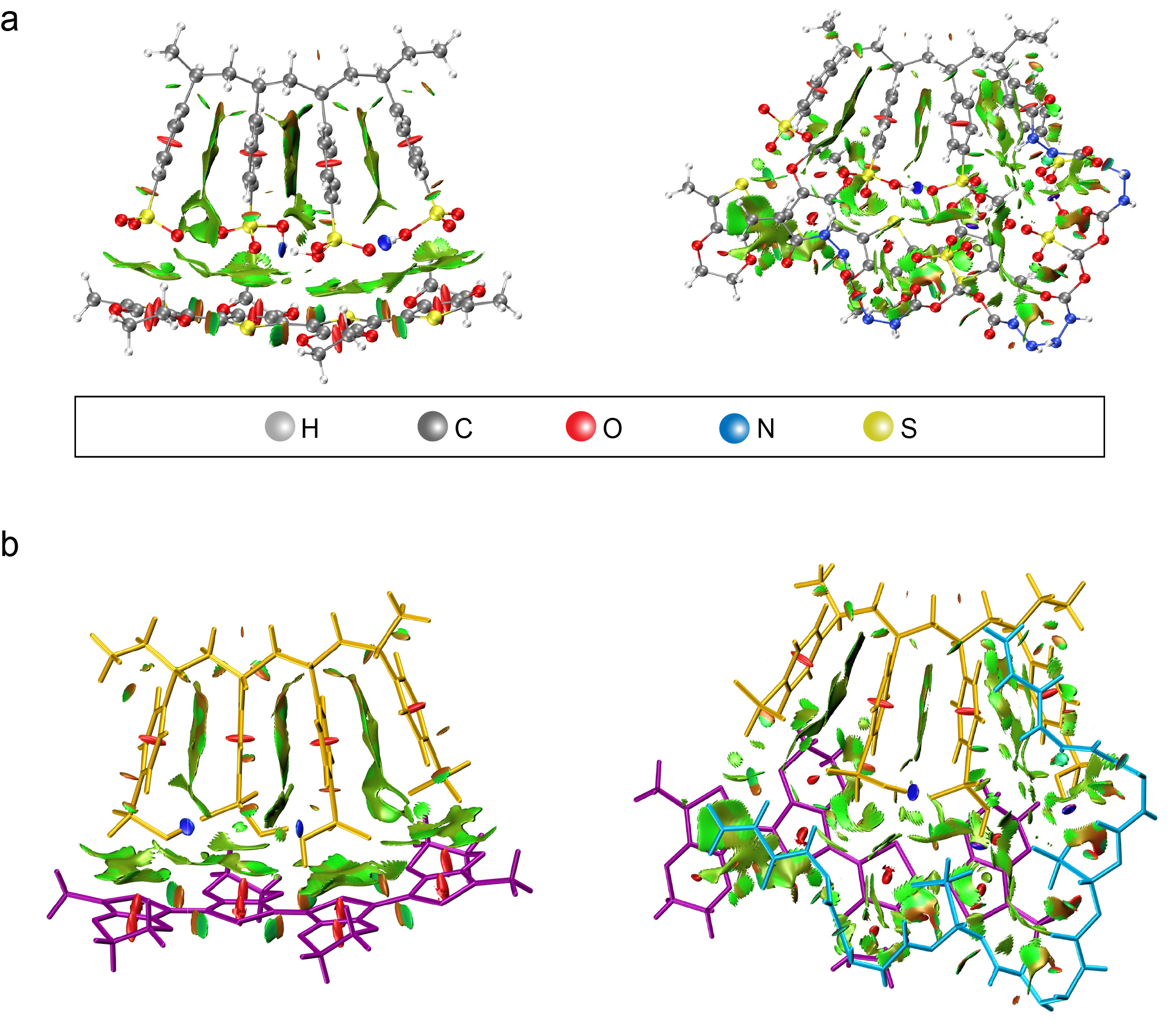


**Figure S8.** Gradient isosurfaces for PEDOT:PSS and PB-CH of RDG mapped by sign (λ2)ρ function. (a) Left: Ball-and-stick model of PEDOT:PSS. Right: Ball-and-stick model of PB-CH. (b) Left: Stick modle of PEDOT:PSS. Right: Stick modle of PB-CH.


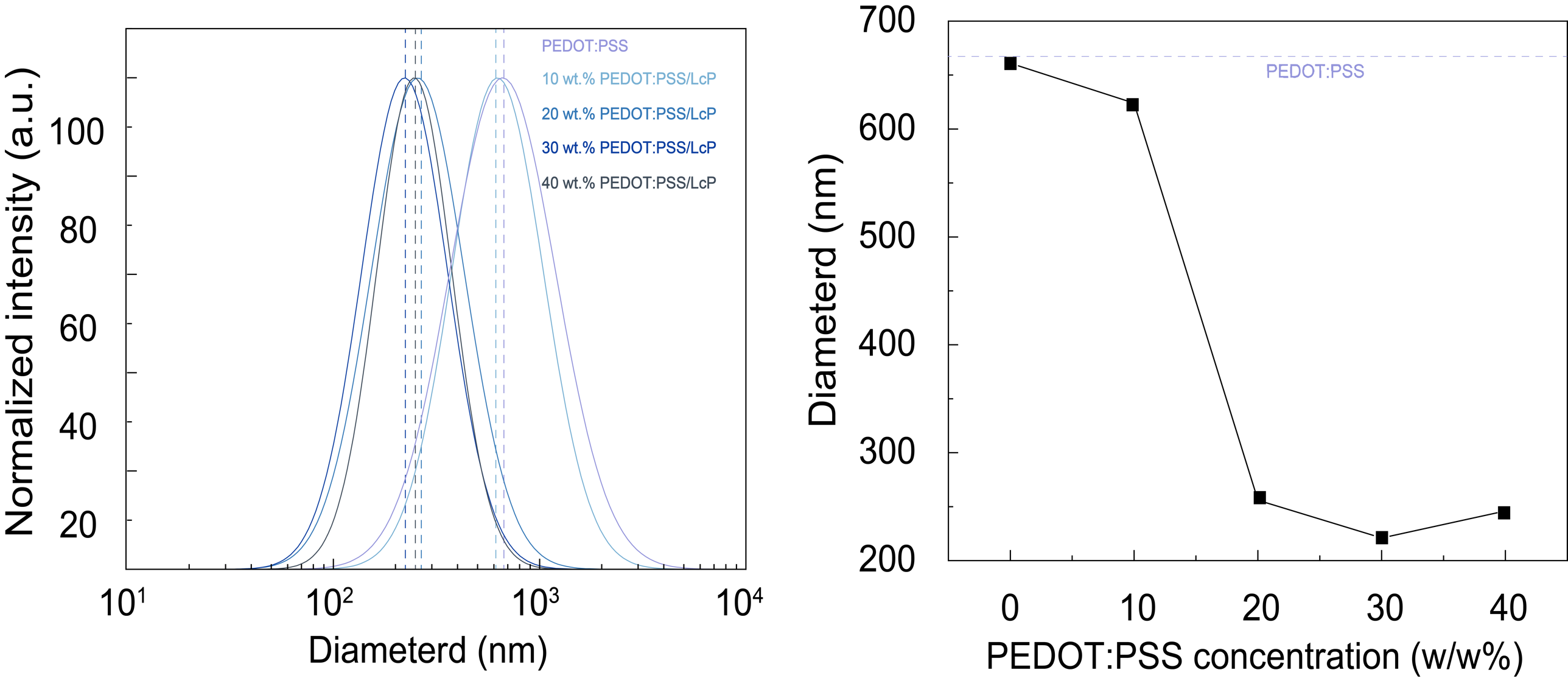


**Figure S9.** Gel particle size distributions of Clevios PH1000 solutions and PB-CH solutions by DLS.


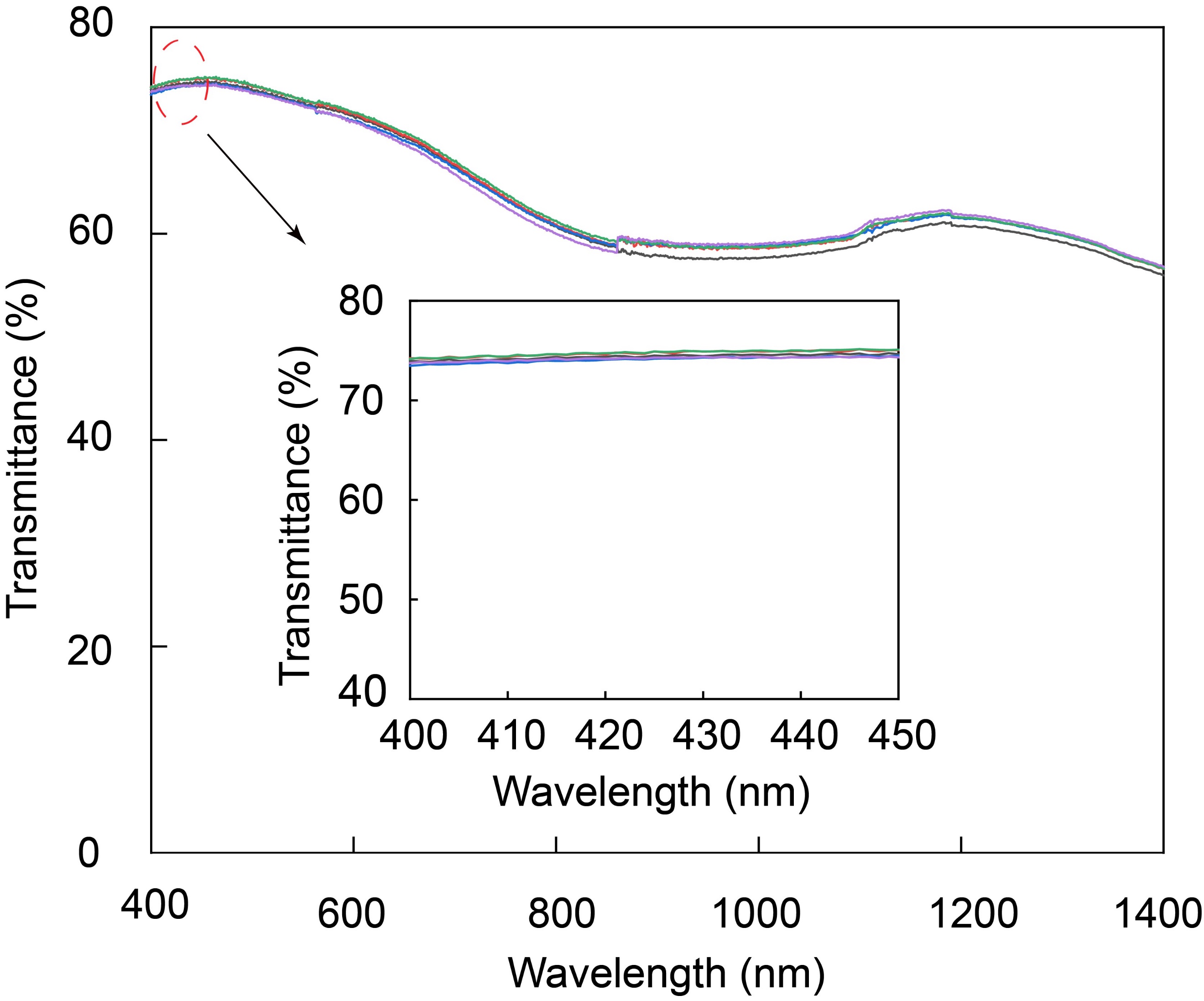


**Figure S10.** Transmittance spectra of PB-CH film at various positions without exposure to 365 nm UV.


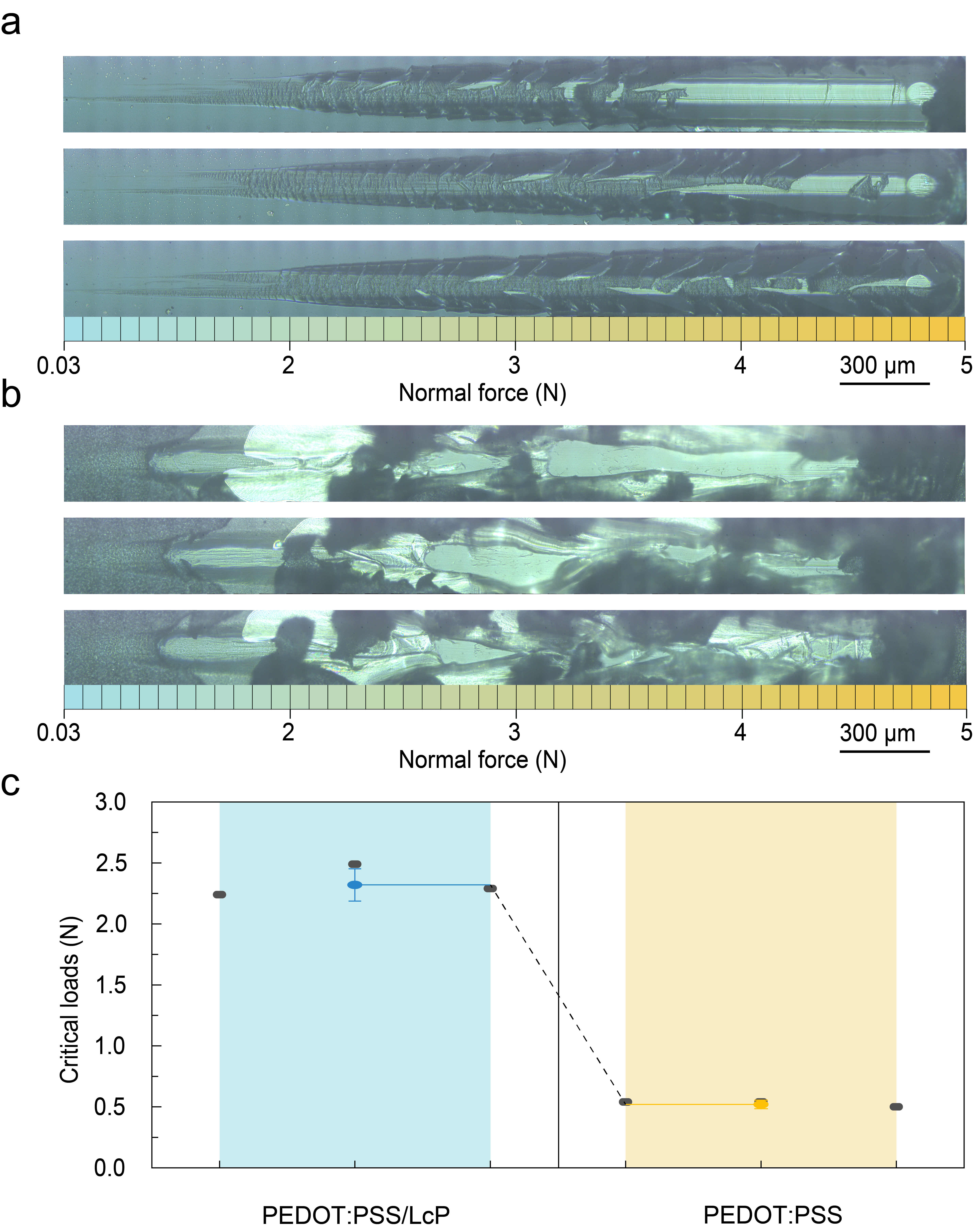


**Figure S11.** Scratch test of PB-CH and PEDOT:PSS. (a) Image obtained after the scratch test of PB-CH, and (b) image obtained after the scratch test of PEDOT:PSS. (c) Critical loads of the UV-cured PB-CH and PEDOT:PSS


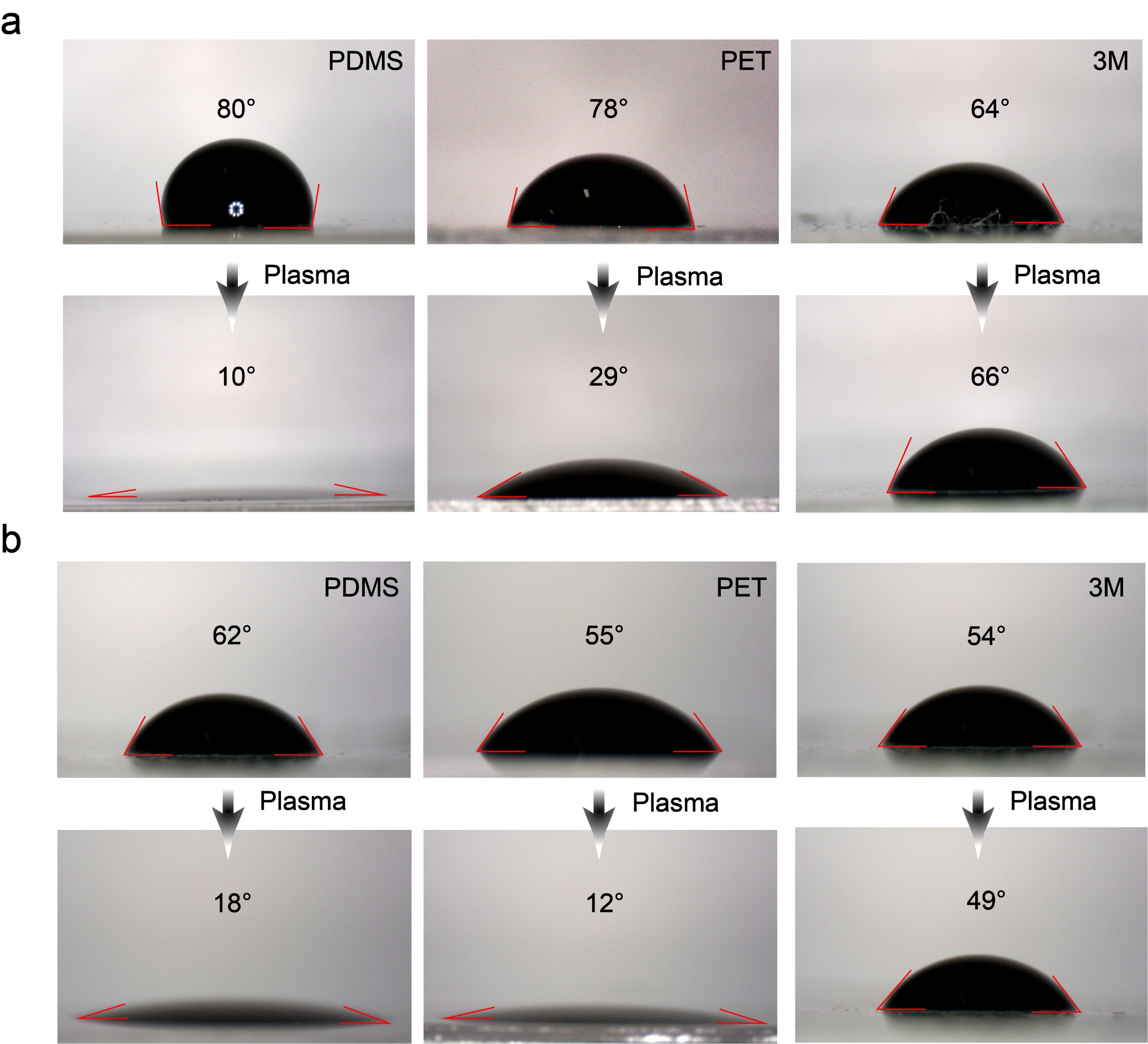


**Figure S12.** The contact angle of PEDO:PSS/LcP soluition on substrates with PH1000 as the control. (a) The contact angle of PH1000 on pristine PDMS, PET, and 3M substrates, respectively, as well as after plasma treatment. (b) The contact angles of photolithographable hydrogel precursor solution on pristine PDMS, PET, and 3M substrates, respectively, as well as after plasma treatment. The enhanced surface wettability of the treated substrate indicates that the plasma has an activating effect on the substrate.


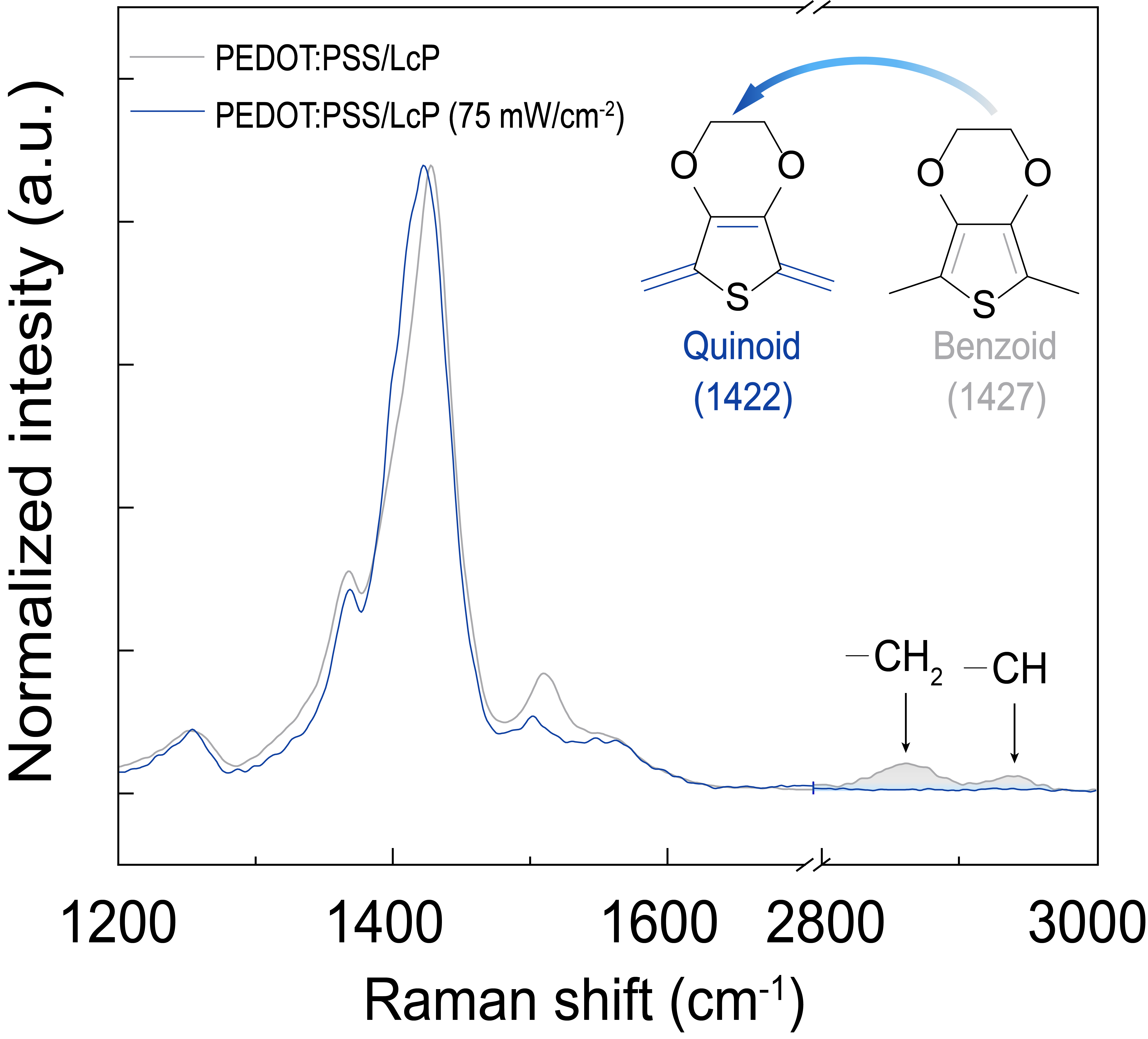


**Figure S13.** Raman spectra of PB-CH film (gray line) and PB-CH film obtained after 365 nm UV irradiation (75 mW cm^−2^, 30 s) (blue line).


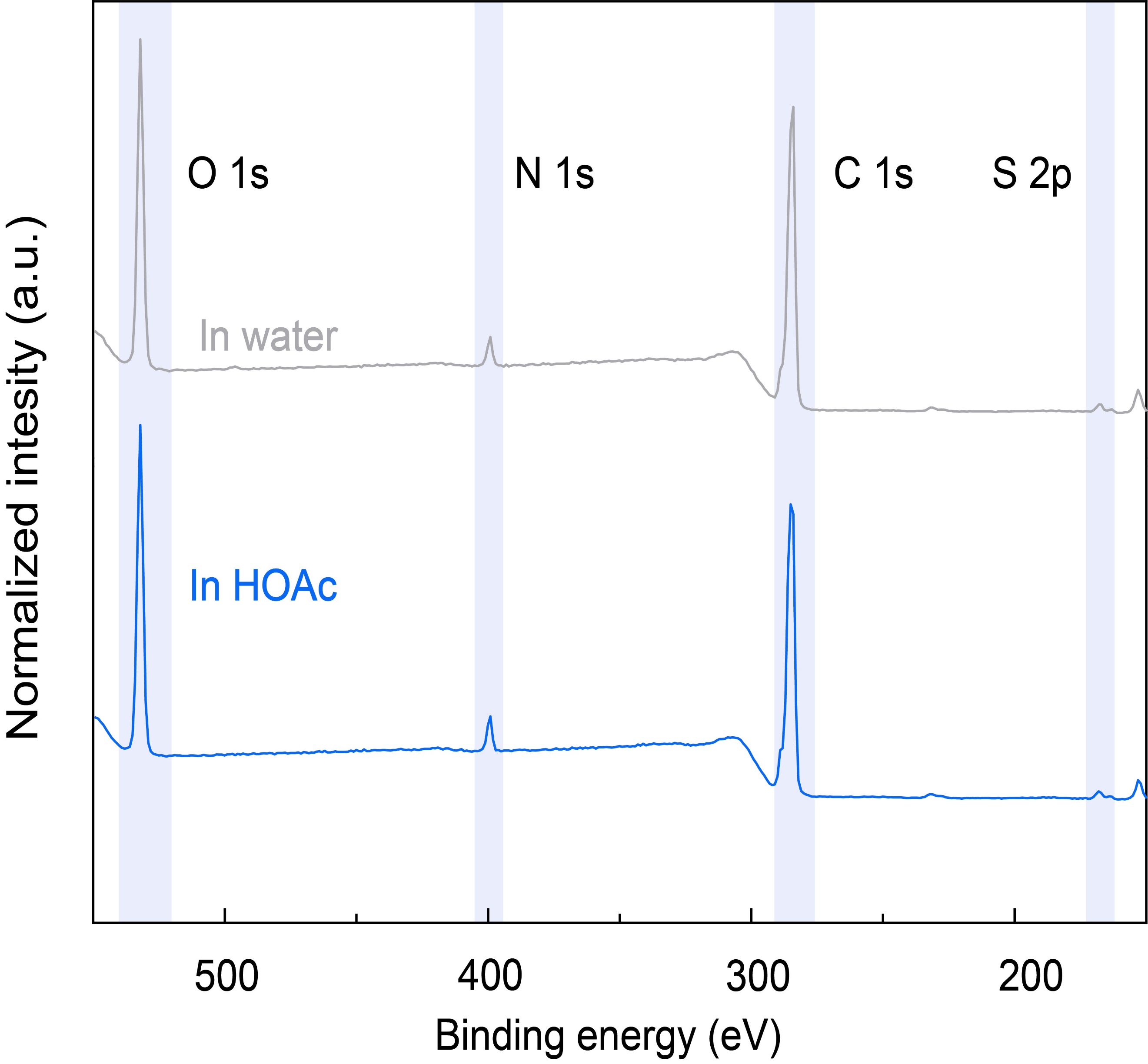


**Figure S14.** Elemental analysis of PEDOT:PSS/LcP and HOAc-treated PB-CH by XPS in the range of 250−550 eV.





**Figure S15.** XPS results for PEDOT:PSS/LcP l and HOAc-treated PB-CH. The deconvolution of XPS peaks was performed between 171 and 162 eV, showing a few increase in the PEDOT to PSS ratio depending on the acetic acid-treated.


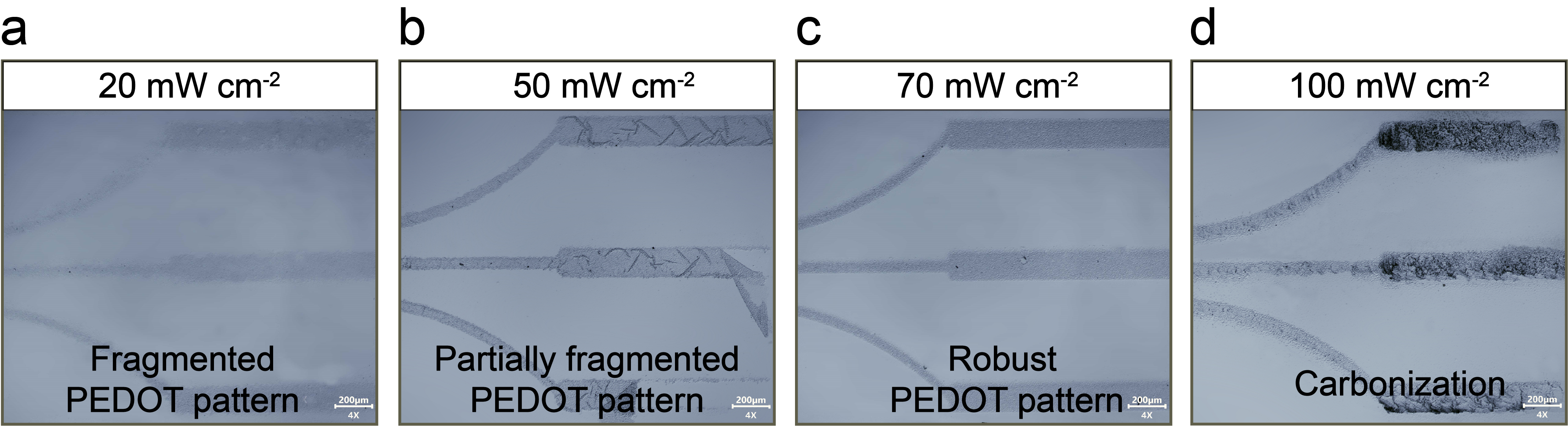


**Figure S16.** The patterning results of PB-CH according to different 365 nm-UV dose. (a), (b) Low power condition. PEDOT:PSS was fragmented when immersed in DI. (c) Optimum condition. The robust PB-CH micropattern was formed. (d) High power condition. PB-CH began to be carbonized.


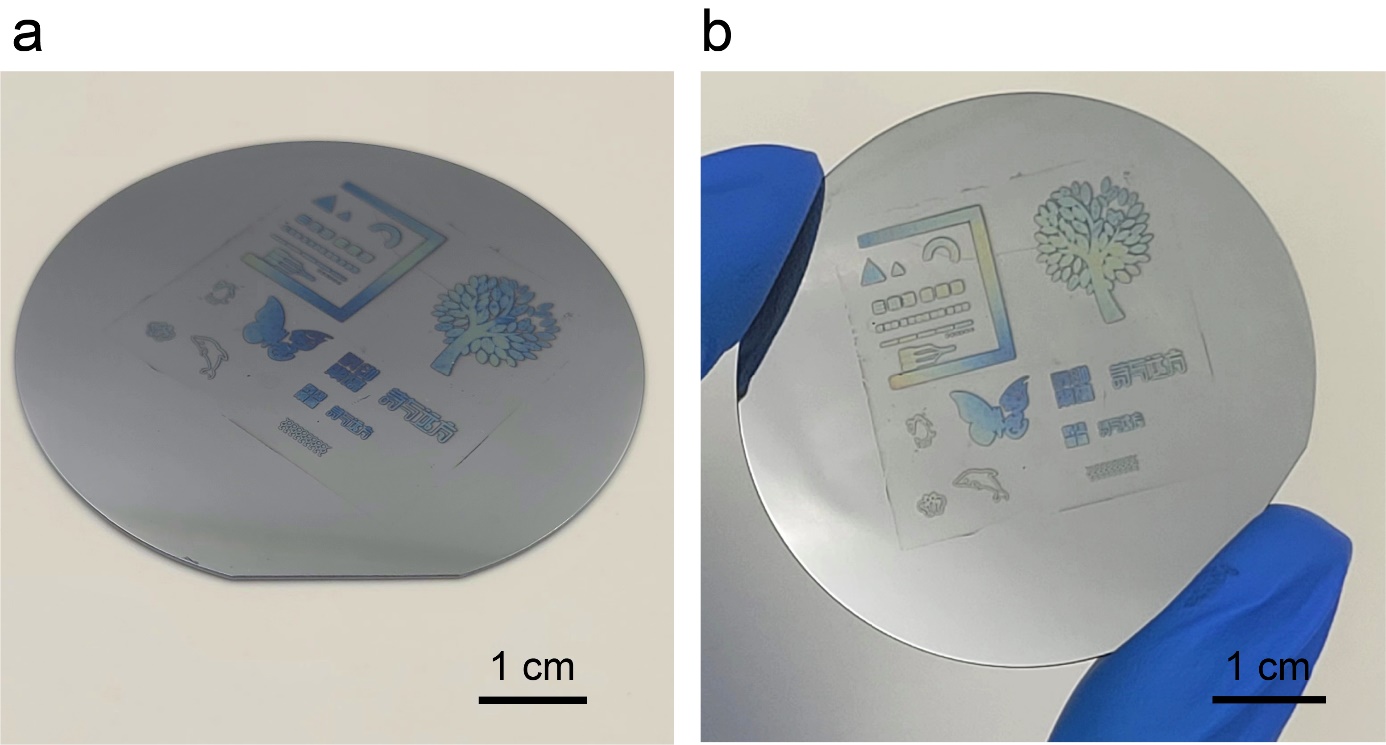


**Figure S17.** Pattern the PB-CH on a Si wafer substrate using direct photolithography.


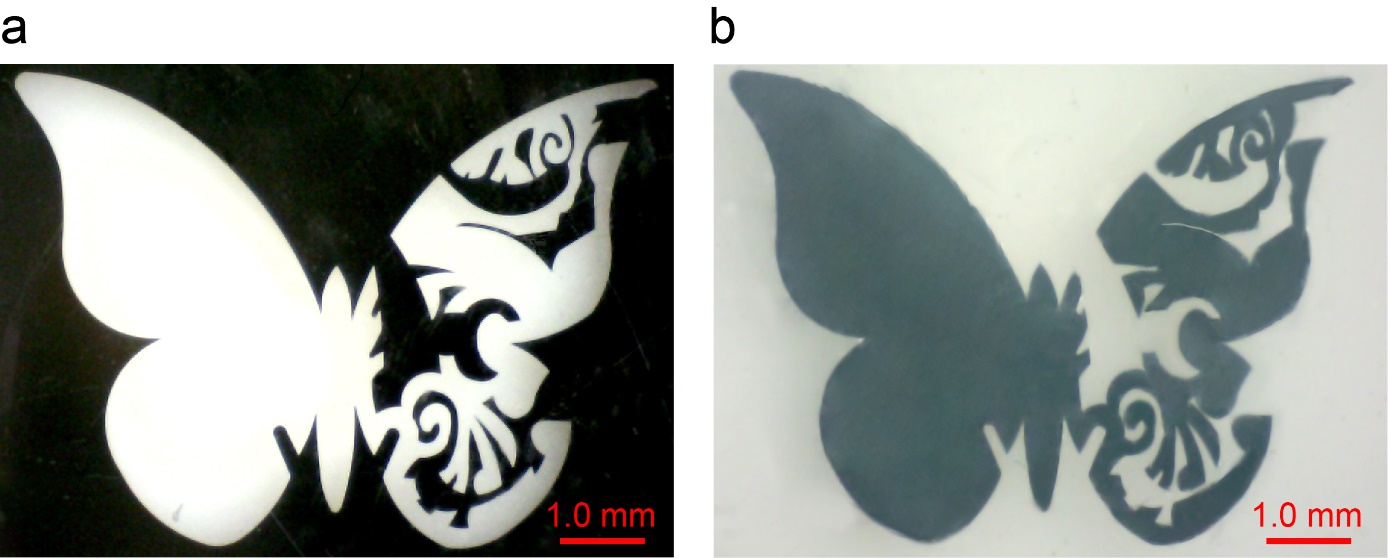


**Figure S18.** Direct lithographically patterend PB-CH. (a) Photomask used in the direct photolithography where the white areas are permeable to 365 nm UV. (b) Optical Image of a PB-CH pattern.


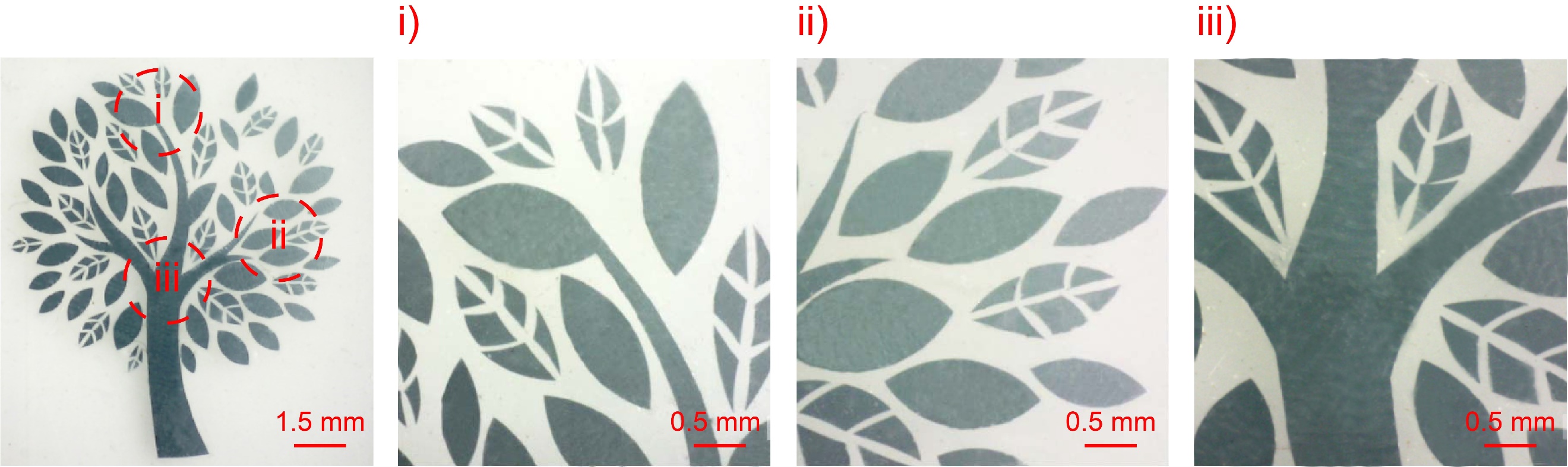


**Figure S19.** Direct lithographically patterned PB-CH pattern along with magnified views of specific regions.


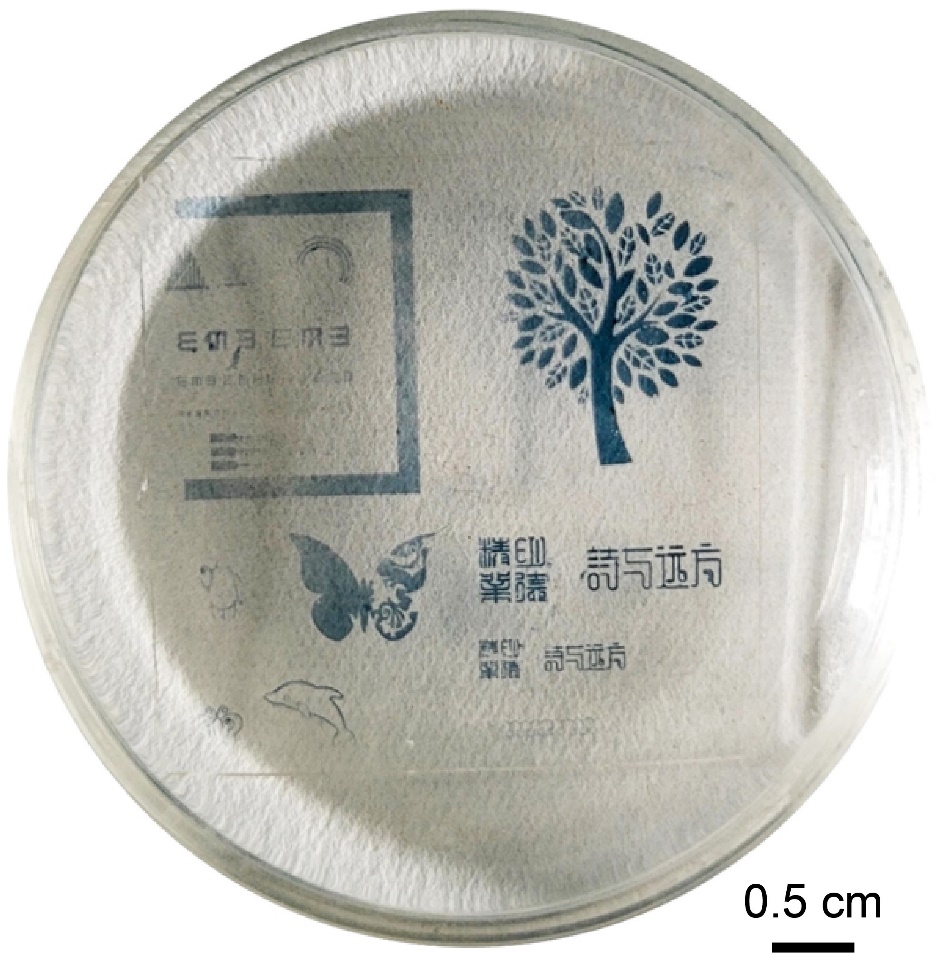


**Figure S20.** Photographs of the PB-CH hydrogel patterns after storage in deionized water for three months.


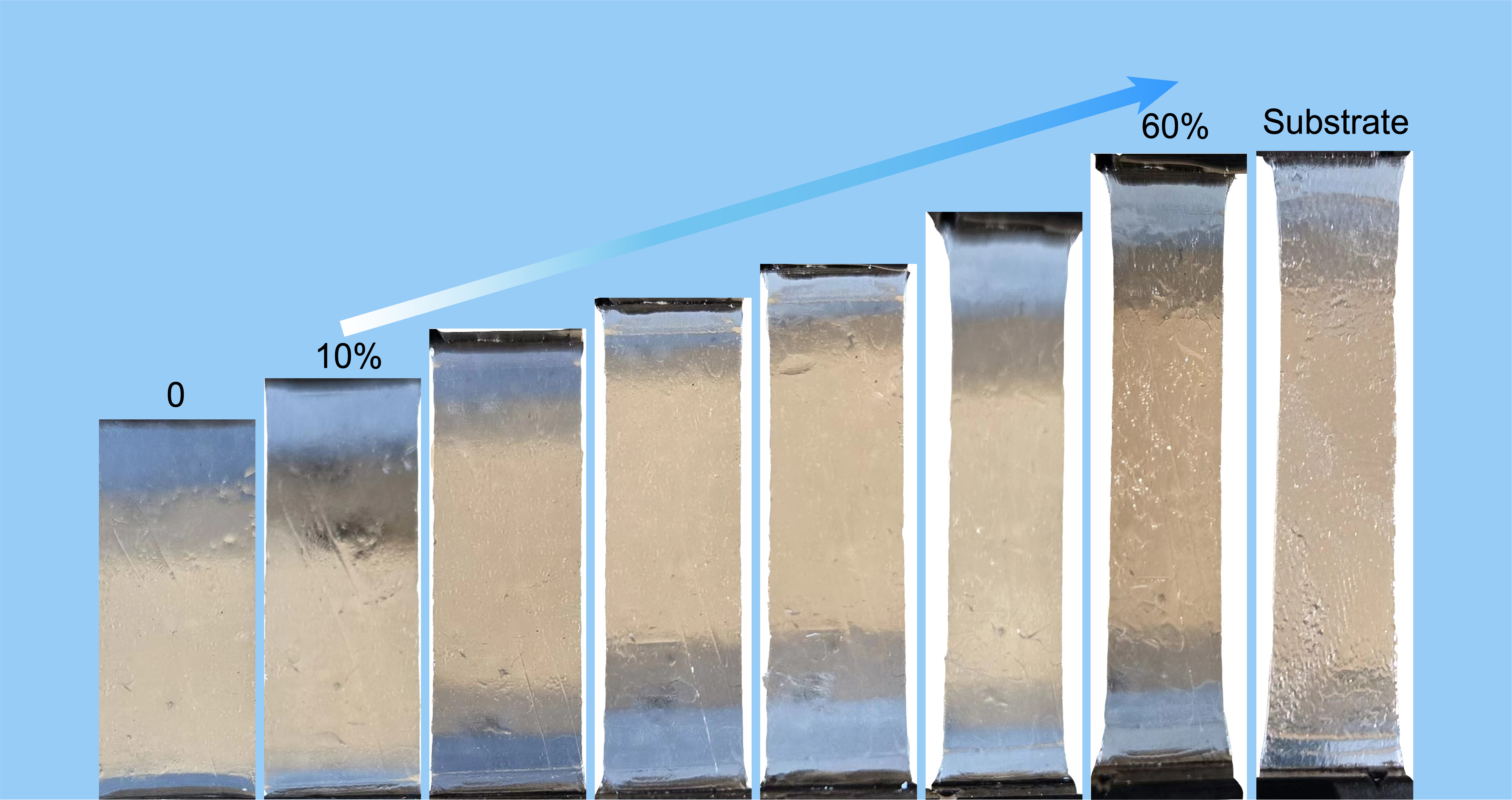


**Figure S21.** Stretching tests were conducted on PB-CH, and optical images were captured to observe crack formation under strain. The hydrogels were prepared by spin coating the photolithographable hydrogel precursor solution onto 3M 300LSE substrates, and then subjected to direct photolithography.

Table S1. Performance of PB-CH varying with different PEDOT:PSS content

| Sample (PB-CH)  PEDOT:PSS / wt.% | Conductivity (S cm^−1^) | Strain (%) | Young’s modulu (KPa) |
| --- | --- | --- | --- |
| 10 | 7.9±2.5 | 85±10 | 7.9±0.27 |
| 20 | 14.8±1.8 | 65±5 | 9.2±0.16 |
| 30 | 30.0±2.4 | 50±5 | 10.6±0.35 |
| 40 | 33.3±1.1 | 30±10 | 90.8±10.5 |

Table S2. Performance comparison between our work and existing works (patterned PEDOT:PSS hydrogels) reported in the literature

| Fabrication  method | Ink  components | Resolution  (μm) | Conductivity (S cm⁻¹) | Stretchability (%) | Reference |
| --- | --- | --- | --- | --- | --- |
| Molding | PEDOT:PSS and PVA | NA | 10 | 150 | 1 |
| Molding | PEDOT:PSS and DBSA | NA | 0.1 | 60 | 2 |
| 3D printing | PEDOT:PSS and PDMS-NH_2_ | 75 | 30.1 | 50 | 3 |
| 3D printing | PEDOT:PSS, PEGDA, and HEA | 100 | NA | NA | 4 |
| Laser treatment | PEDOT:PSS and AuNP | 6 | 670 | NA | 5 |
| Laser treatment | PEDOT:PSS | 5 | 101 | NA | 6 |
| Photolithography | PEDOT:PSS and IL | 5 | 47 | 20 | 7 |
| Photolithography | PEDOT:PSS, SBMA, and PEGDA | 50 | 6 | 290 | 8 |
| Photolithography | PEDOT:PSS, and LcP | 5 | 30 | 50 | This work |

Reference

[1] S. Zhang, Y. Chen, H. Liu, Z. Wang, H. Ling, C. Wang, J. Ni, B. Çelebi-Saltik, X. Wang, X. Meng, H.J. Kim, A. Baidya, S. Ahadian, N. Ashammakhi, M.R. Dokmeci, J. Travas-Sejdic, A. Khademhosseini, Room-temperature-formed PEDOT:PSS hydrogels enable injectable, soft, and healable organic bioelectronics, Adv. Mater. 2020, 32, 1904752, https://doi.org/10.1002/adma.201904752.

[2] G. Li, K. Huang, J. Deng, M. Guo, M. Cai, Y. Zhang, C.F. Guo, Highly conducting and stretchable double-network hydrogel for soft bioelectronics, Adv. Mater. 2022, 34, 2200261, https://doi.org/10.1002/adma.202200261.

[3] X. Xie, Z. Xu, X. Yu, H. Jiang, H. Li, W. Feng, Liquid-in-liquid printing of 3D and mechanically tunable conductive hydrogels, Nat. Commun. 2023, 14, 4289, https://doi.org/10.1038/s41467-023-40004-7.

[4] N. Lopez-Larrea, M. Criado-Gonzalez, A. Dominguez-Alfaro, N. Alegret, I.d. Agua, B. Marchiori, D. Mecerreyes, Digital light 3D printing of PEDOT-based photopolymerizable inks for biosensing, ACS Appl. Polym. Mater. 2022, 4, 6749, https://doi.org/10.1021/acsapm.2c01170.

[5] D. Won, J. Kim, J. Choi, H. Kim, S. Han, I. Ha, J. Bang, K. Kyu Kim, Y. Lee, T. Kim, J. Park, C. Kim, S. Hwan Digital selective transformation and patterning of highly conductive hydrogel bioelectronics by laser-induced phase separation, Sci. Adv. 2022, 8, eabo3209, https://doi.org/10.1126/sciadv.abo3209.

[6] X. Wang, Y. Feng, K. Sun, N. Chai, B. Mai, S. Li, X. Chen, W. Zhao, Q. Zhang, Ultrafast laser-induced excellent thermoelectric performance of PEDOT:PSS films, Energy Environ. Mater. 2024, 7, e12650, https://doi.org/10.1002/eem2.12650.

[7] Y. Liu, J. Liu, S. Chen, T. Lei, Y. Kim, S. Niu, H. Wang, X. Wang, A.M. Foudeh, J.B.H. Tok, Z. Bao, Soft and elastic hydrogel-based microelectronics for localized low-voltage neuromodulation, Nat. Biomed. Eng. 2019, 3, 58, https://doi.org/10.1038/s41551-018-0335-6.

[8] M. Yang, P. Chen, X. Qu, F. Zhang, S. Ning, L. Ma, K. Yang, Y. Su, J. Zang, W. Jiang, T. Yu, X. Dong, Z. Luo, Robust neural interfaces with photopatternable, bioadhesive, and highly conductive hydrogels for stable chronic neuromodulation, ACS Nano. 2023, 17, 885, https://doi.org/10.1021/acsnano.2c04606.
